# Low-burden AI approach for cross-national early identification of cognitive impairment using real-world questionnaire response behaviours

**DOI:** 10.1038/s41467-026-76071-9

**Published:** 2026-07-28

**Authors:** Hongxin Gao, Stefan Schneider, Jenny Harris, Raymond Hernandez, Doerte U. Junghaenel, Arie Kapteyn, Pey-Jiuan Lee, Arthur Stone, Elizabeth Zelinski, Danny Maupin, Bart Orriens, Haomiao Jin

**Affiliations:** 1 School of Health Sciences, Faculty of Health and Medical Sciences, University of Surrey, Guildford, UK; 2 Center for Self-Report Science, University of Southern California, Los Angeles, CA USA; 3 Department of Psychology, University of Southern California, Los Angeles, CA USA; 4 Center for Economic and Social Research, University of Southern California, Los Angeles, CA USA; 5 Leonard Davis School of Gerontology, University of Southern California, Los Angeles, CA USA

**Keywords:** Dementia, Human behaviour, Cognitive ageing

## Abstract

Under-recognition of cognitive impairment remains a major global public health challenge, particularly in low- and middle-income countries. Many cases go unrecognised because conventional cognitive assessment is resource-intensive and difficult to scale across diverse populations. Here we show that easily captured indicators of reduced survey response quality, derived from how older adults answer routine psychosocial questionnaires, can identify cognitive impairment risk across countries. Using data from 45,604 older adults across five population-based ageing cohorts, we develop a cross-nationally generalisable machine-learning pipeline based on a tabular foundation model. Especially, the model jointly trained across the United States, England, India and Mexico outperforms country-specific models, achieving up to a 0.12 absolute gain in the area under the receiver operating characteristic curve (approximately 20% relative) in an external validation cohort from China. We also identify a cross-national implicit feature transfer phenomenon, in which cohorts lacking certain predictors benefit from joint training with cohorts in which these predictors are present. Decision-curve analyses indicate consistent potential public health benefit across countries by supporting population-level prioritisation of higher-risk subgroups. This low-burden approach offers an equitable and scalable strategy for generating population- and community-level evidence on cognitive impairment risk in culturally diverse and resource-constrained settings.

## Abstract (web summary)

This study shows that AI can use patterns in how older adults answer routine questionnaires to identify cognitive impairment risk across five countries, supporting low-burden population-level cognitive health monitoring and research.

## Introduction

Cognitive impairment, encompassing mild cognitive impairment (MCI) and dementia, remains a major global public health challenge of the 21st century. Over 57 million people are currently estimated to be affected worldwide, a figure projected to reach 139 million by 2050, with most new cases arising in low- and middle-income countries (LMICs)^1,2^. Even more concerning is the substantial under-recognition: in high-income countries, only 20–50% of cases are recognised in primary care^2^, while in some LMICs this proportion can be less than 10%^3,4^. The recent approval of medications that can slow cognitive decline, such as Lecanemab and Donanemab^5,6^, heightens the urgency of timely identification, as these therapies are most effective in the early stages of cognitive impairment. This need has also been recognised in international public health policy frameworks, including recent World Health Organisation (WHO) initiatives that call for scalable, low-burden and culturally generalisable approaches to population-level surveillance and early detection of cognitive impairment^3^.

Current screening approaches typically rely on standardised cognitive tests, such as the Mini-Mental State Examination (MMSE) and Montreal Cognitive Assessment (MoCA), or biomarker-based laboratory tests (e.g., neuroimaging, fluid biomarkers). These approaches face several challenges that limit their widespread use in routine public health practice. First, they are resource-intensive: cognitive assessments typically require trained personnel and in-person administration^7,8^, while biomarker-based tools are invasive and dependent on clinical infrastructure^9,10^, limiting scalability in resource-constrained settings. Second, their cross-national validity is often limited. Most cognitive screening instruments have been developed in high-income or single-country contexts, frequently introducing bias related to educational and linguistic differences^11–13^. Applying these tools directly across diverse cultural contexts risks systematic biases, potentially exacerbating global cognitive health inequalities^14,15^. Similar concerns have also been raised in data-driven and machine learning–based cognitive prediction research, which has highlighted the importance of involving diverse populations from various countries or regions in risk modelling^16^. Third, integrating cognitive risk screening into existing public health and healthcare workflows adds a substantial burden, increasing frontline staff workload and healthcare resource pressure, particularly in LMICs^17–19^.

These limitations motivate a complementary, process- and behaviour-centred approach. Rather than additional cognitive testing or biomarker-based assessments, we leverage readily obtained behavioural markers derived from routine questionnaire responses. Large-scale epidemiological survey studies of ageing populations worldwide have accumulated vast quantities of real-world questionnaire data, offering a practical yet underexplored opportunity to develop lower-burden approaches for identifying cognitive impairment across diverse populations. In these surveys, how respondents answer may be as informative as what they answer. A growing body of evidence indicates that readily observable response behaviours—such as item non-response or skipping, inconsistent answering and aberrant response patterns, collectively termed low-quality response (LQR) indicators—can capture subtle changes in cognitive functioning^20–25^. For researchers with experience in large-scale survey studies, such signals are far from unfamiliar; yet they have typically been treated as noise or data-quality artefacts to be discarded rather than utilised as informative predictors, leaving a potentially rich source of cognitive-health information largely untapped.

LQR indicators present several potential advantages for population-level cognitive risk identification: (1) Ease of acquisition and scalability, as they can be automatically extracted from existing or newly developed brief questionnaires without additional burden for survey takers or assessors^23,26^; (2) Reduced stigma and psychological burden, as LQR leverages response behaviours from routinely administered questionnaires rather than tasks explicitly labelled as cognitive tests, thereby avoiding the overt testing frame and evaluative threat associated with formal cognitive assessment; and (3) Cross-national predictive potential, supported by a multinational meta-analysis demonstrating stable associations between LQR indicators and cognitive function across diverse cultural contexts^25^. From a public health perspective, if harnessed systematically, these routinely captured response behaviours could support population-level identification of cognitive impairment risk in settings where formal cognitive assessment is not feasible, while also improving cross-national comparability for population-level inference and surveillance^3^.

**Fig. 1:**
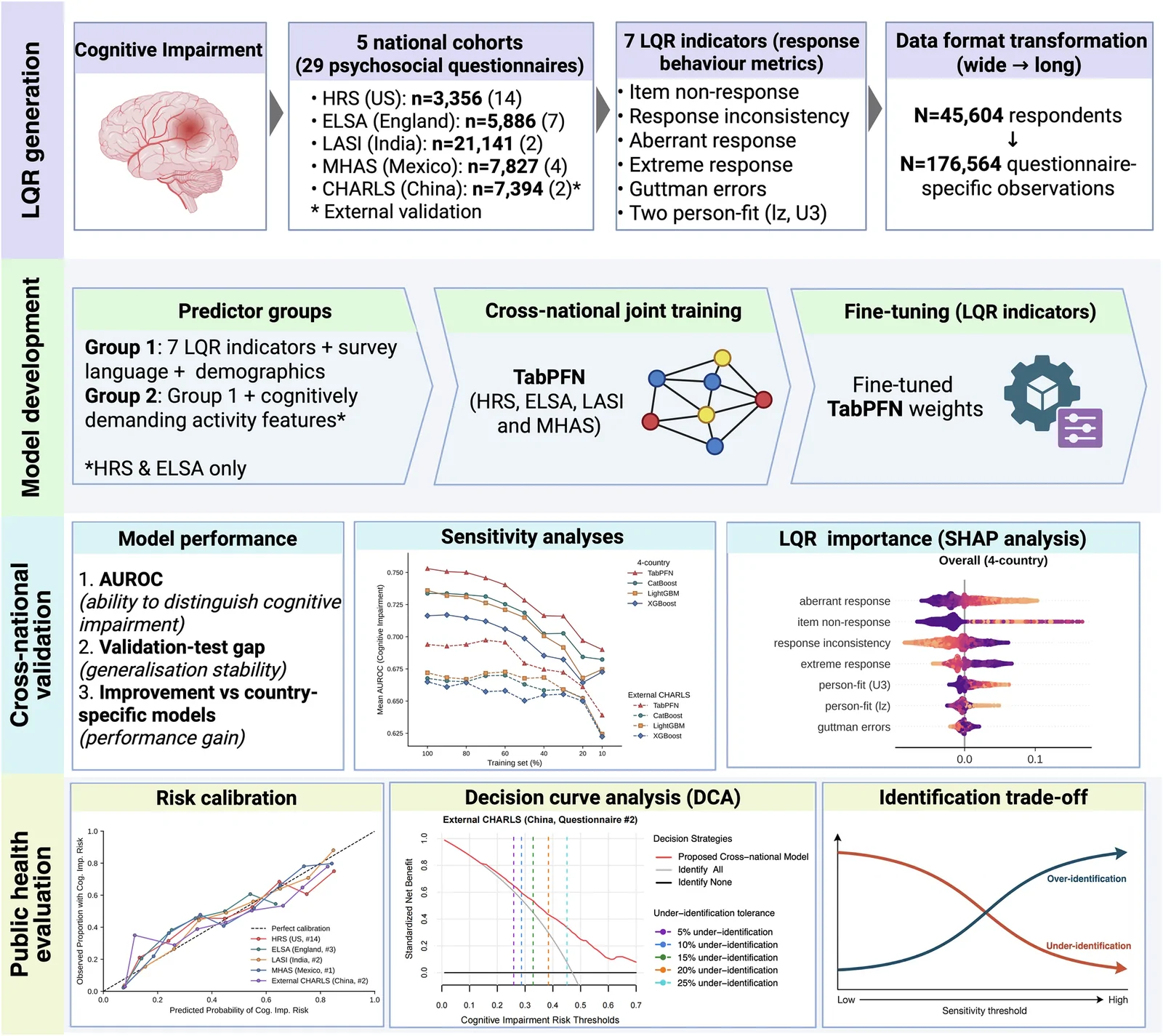
Low-burden AI pipeline for cognitive impairment risk identification. (1) Low-quality response (LQR) indicators generated from questionnaire responses of 45,604 respondents aged ≥65 from five national cohorts (HRS [US], ELSA [England], LASI [India], MHAS [Mexico], CHARLS [China; external validation]). (2) Cross-national predictive models developed using Tabular Prior-data Fitted Network (TabPFN) with two predictor groups: group 1 (seven LQR indicators, survey language, and demographics [age, sex, and education level]); and group 2 (group 1 plus cognitively demanding activity features, available only in HRS [email use, driving status] and ELSA [internet/email use and frequency]). (3) Cross-national evaluation of the model for cognitive impairment based on discrimination, generalisation, robustness, and feature importance analysis (SHapley Additive exPlanations [SHAP]). (4) Potential public health evaluation of model predictions, including risk calibration, decision curve analysis (DCA), and a conceptual illustration of under-identification and over-identification trade-offs. HRS Health and Retirement Study, ELSA English Longitudinal Study of Ageing, LASI Longitudinal Ageing Study in India, MHAS Mexican Health and Ageing Study, CHARLS China Health and Retirement Longitudinal Study, AUROC Area under the receiver operating characteristic curve. Created in BioRender. Gao, H. (2026) https://BioRender.com/94si9ut.

Despite growing evidence that survey response behaviours capture meaningful variation in cognitive functioning, these signals have not yet been developed into scalable, cross-nationally generalisable tools for identifying the risk of cognitive impairment. Our previous formative work demonstrated the concept of this approach in a single developed-country setting^26^; however, LMIC populations have not been systematically included in model development or validation, limiting its applicability in a global public health context. Here, we address this gap by leveraging population-based survey data from 45,604 older adults spanning three continents and five countries—the United States, England, India, Mexico, and China—to develop and validate a low-burden, cross-nationally generalisable machine-learning pipeline for identifying cognitive impairment risk (Fig. 1). We evaluate its performance across these diverse populations and examine its potential as a scalable approach for international public health evidence generation. More broadly, this approach reframes routine survey completion as a source of cognitive-health information, rather than merely a vehicle for self-report data.

## Results

Figure 1 provides an overview of our analytical pipeline. Briefly, we first derived seven LQR indicators from questionnaire data collected across five population-based ageing survey studies in the United States, England, India, Mexico, and China, obtained via the Gateway to Global Ageing Data^27^. These LQR indicators were combined into two primary predictor sets: Group 1 (LQR indicators, survey language, and demographics) and Group 2 (Group 1 plus cognitively demanding activity features, including internet/email use and driving status, available only in the US and England cohorts). Prediction outcomes were defined using Bayesian multilevel latent class analysis based on harmonised cognitive factor scores from Harmonised Cognitive Assessment Protocol (HCAP) sub-studies conducted within each country^27,28^, resulting in three cognitive status categories (cognitively normal, MCI, dementia). Cross-national predictive models were then developed using the Tabular Prior-data Fitted Network (TabPFN) to perform three-class classification^29^, with cognitive impairment (combining predicted probabilities for MCI and dementia) as the primary outcome, followed by additional fine-tuning analyses. Comprehensive evaluation of the pipeline included generalisability, robustness and interpretability.

### Respondent characteristics across five national ageing cohorts

The study sample comprised participants from the Health and Retirement Study (HRS, US; *N*  =  3356, mean [SD] age = 75.7 [7.2] years, 60.3% female), English Longitudinal Study of Ageing (ELSA, England; *N*  =  5886, mean [SD] age = 75.2 [7.0] years, 56.0% female), Longitudinal Ageing Study in India (LASI, India; *N*  =  21,141, mean [SD] age = 72.2 [6.6] years, 50.7% female), Mexican Health and Ageing Study (MHAS, Mexico; *N*  =  7827, mean [SD] age = 73.8 [6.8] years, 53.4% female), and China Health and Retirement Longitudinal Study (CHARLS, China; external validation; *N*  =  7394, mean [SD] age = 72.4 [6.3] years, 51.4% female). Overall, 72.3% of respondents had less than upper secondary education, with cohort-specific proportions as follows: 16.1% in HRS, 30.0% in ELSA, 79.3% in LASI, 89.8% in MHAS, and 93.1% in CHARLS. Detailed characteristics of the study sample are presented in Supplementary Table 1.

Respondents within each country completed a range of psychosocial questionnaires (14 in HRS, 7 in ELSA, 4 in MHAS, and 2 each in LASI and CHARLS; Supplementary Table 2). This yielded a long-format dataset of 176,564 questionnaire-specific observations (*N* = 45,604 respondents), among which 35% were randomly selected to form two independent test sets for subsequent model evaluation: an internal test set consisting of respondents from the four-country dataset (HRS, ELSA, LASI, MHAS; *N* = 13,376 respondents, 56,637 observations), and an external validation set consisting solely of respondents from CHARLS (*N* = 2588 respondents, 5176 observations).

### Analysis of LQR indicators

To examine the cross-national predictive value of LQR indicators, we first compared the predictive performance of the LQR predictors alone (trained jointly on HRS, ELSA, LASI, and MHAS using default TabPFN weights; 7 LQR indicators and survey language, Supplementary Tables 3 and 4) with demographic variables (age, sex, education) for identifying cognitive impairment. Using only the LQR predictors, AUROC values ranged from 0.54 to 0.78 when evaluated separately for each of the 29 psychosocial questionnaires and countries (Supplementary Table 5), with average AUROC of 0.67 (four-country joint-training) and 0.66 (external validation CHARLS). Demographic variables alone achieved an average AUROC of 0.74 and 0.64, respectively. We observed that these two different types of predictors were complementary rather than mutually competitive: that is, although demographic variables showed slightly better predictive performance in the overall test sample, their ability to discriminate cognitive impairment dropped substantially (average AUROC: four-country joint-training = 0.48, external validation CHARLS = 0.60) within known at-risk demographic subgroups (e.g., those aged ≥80 years with less than upper secondary education). In contrast, the LQR predictors maintained stable predictive performance within the at-risk subgroup (average AUROC: four-country joint-training = 0.64, external validation CHARLS = 0.65).

Therefore, in subsequent analyses, two predictive models were developed to comprehensively evaluate cross-national performance: Model I, which uses LQR indicators, survey language and demographics as predictors, due to their complementary contribution to prediction; and Model II using LQR indicators, survey language, demographics, and four variables indicating engagement in certain cognitively demanding activities in daily life as predictors. These additional predictors were available only in the HRS (email use, driving status) and ELSA (internet/email use and frequency; Supplementary Table 6), allowing us to examine whether features learned in one setting can improve prediction in others (cross-national implicit feature transfer).

### Predictive performance of the cross-national model for cognitive impairment

**Fig. 2:**
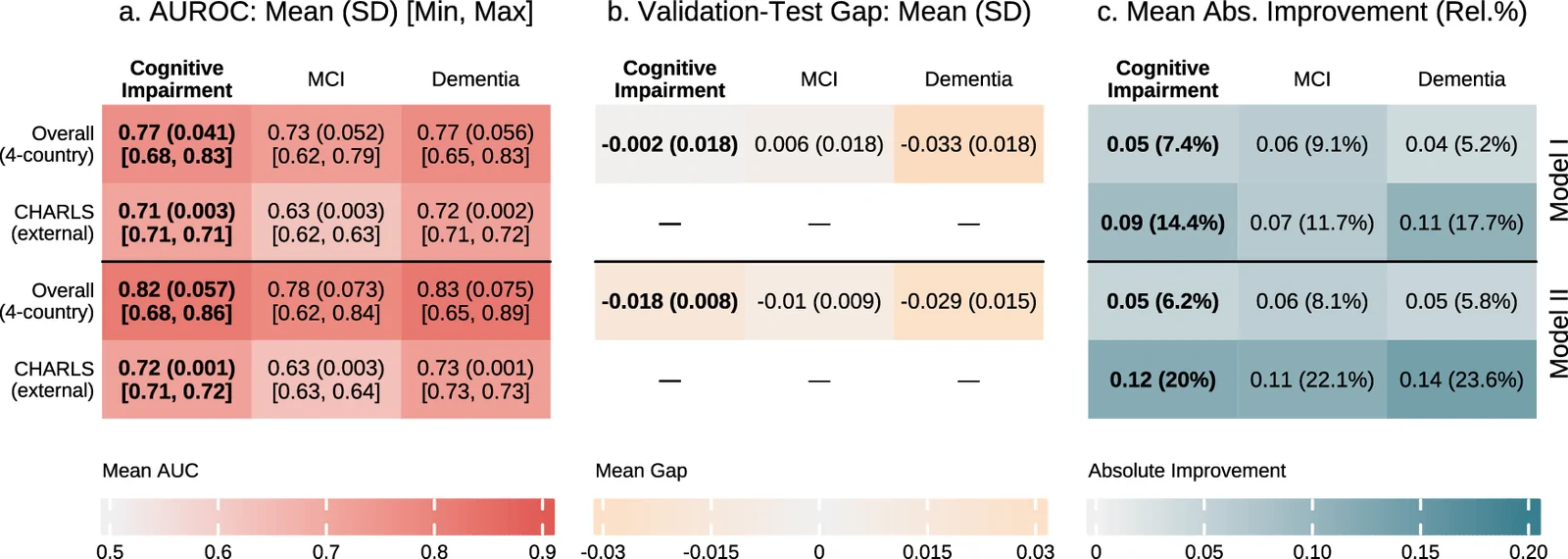
Cross-national model discrimination, generalisation gap, and improvements compared to country-specific models in identifying current cognitive impairment risk. **a** AUROC (area under the receiver operating characteristic curve) performance of cross-national models for cognitive impairment risk (cognitive impairment [mild cognitive impairment (MCI) and dementia] vs. cognitively normal), MCI risk (MCI vs. cognitively normal), and dementia risk (dementia vs. cognitively normal). **b** Validation–test AUROC gap, indicating generalisation stability. Gaps were not applicable for external CHARLS validation due to the absence of a validation set. **c** Improvement of cross-national compared with country-specific models: absolute AUROC improvement and relative improvement (%). All metrics were calculated at the questionnaire level. Model I = low-quality response (LQR) indicators, survey language and demographics; Model II = Model I plus cognitively demanding activity features. HRS and ELSA contributed activity features to Model II. Independent test sets (35%): four-country, *N* = 13,376 respondents (56,637 questionnaire-specific observations); external CHARLS, *N* = 2588 respondents (5176 questionnaire-specific observations). CHARLS China Health and Retirement Longitudinal Study, ELSA English Longitudinal Study of Ageing, HRS Health and Retirement Study, LASI Longitudinal Ageing Study in India, MHAS Mexican Health and Ageing Study. Abs. absolute, Max maximum, Min minimum, Rel. relative, SD standard deviation. Source data are provided as a Source Data file.

Figure 2 summarises the generalisation performance of the cross-national models trained jointly on HRS, ELSA, LASI, and MHAS data. Using LQR indicators, survey language, and demographics (Model I), the model achieved an average AUROC of 0.77 for cognitive impairment, and above 0.70 for MCI and dementia. After incorporating activity features available only in HRS and ELSA (Model II), the average AUROC further improved to 0.82, with performance gains extending to cohorts lacking these features. Regardless of predictor selection, the validation-test gap remained minimal (within ±0.02; Fig. 2b), indicating robust generalisation. Sensitivity analyses across 100 random data-splitting repetitions further supported robustness, with predictive performance consistently remaining within ±1 SD (Supplementary Fig. 1a).

Compared with country-specific models (Fig. 2c), the cross-national joint training strategy exhibited better predictive power for cognitive impairment, achieving relative AUROC improvements of up to 7.4%. The performance gains were even greater in external validation on CHARLS, with relative improvements of 14–20% (absolute increases of 0.09–0.12). Notably, this advantage persisted even compared with models trained exclusively on CHARLS data (Supplementary Fig. 2), highlighting strong cross-national generalisability despite CHARLS data not being used in training. Moreover, the proposed model consistently outperformed three classic ensemble models (CatBoost, LightGBM, and XGBoost; trained on the same cross-national data) and maintained a mean AUROC above 0.7 even at only 25% of the original training data, suggesting robust performance under limited data conditions (Supplementary Fig. 1b).

Importantly, when adding activity features (Model II), predictive performance improved not only in cohorts with available activity features (HRS and ELSA), but also in all three LMIC cohorts lacking these features (paired *t*-test across eight questionnaires from LASI, MHAS, and CHARLS: *p* = 0.003; largest improvement: +2.1% in the LASI Satisfaction with Life questionnaire; Supplementary Table 5). This implicit feature transfer phenomenon, in which a cohort gains predictive benefit from features not collected in its own data, was observed only in the jointly trained TabPFN model, but not in country-specific models or the three ensemble learning methods.

To examine the robustness of prospective prediction, we conducted an analysis using earlier-wave LQR indicators where available to predict outcomes at later waves (Supplementary Table 7). Predictive performance remained broadly comparable to concurrent analyses, with average AUROC of 0.74 (Model I) and 0.82 (Model II) in the internal four-country test set and 0.68–0.69 in external validation on CHARLS. These findings indicate that the LQR-based cross-national model maintains robust prospective predictive performance.

We also fine-tuned the default TabPFN weights and observed modest but consistent improvements in predictive performance, with gains in over 92% of questionnaires in the internal four-country test set and across all questionnaires in the external validation (Supplementary Table 5 and Supplementary Note 1). The fine-tuned weights were applied in subsequent analyses.

### Interpretation of LQR indicators across countries and subgroups

**Fig. 3:**
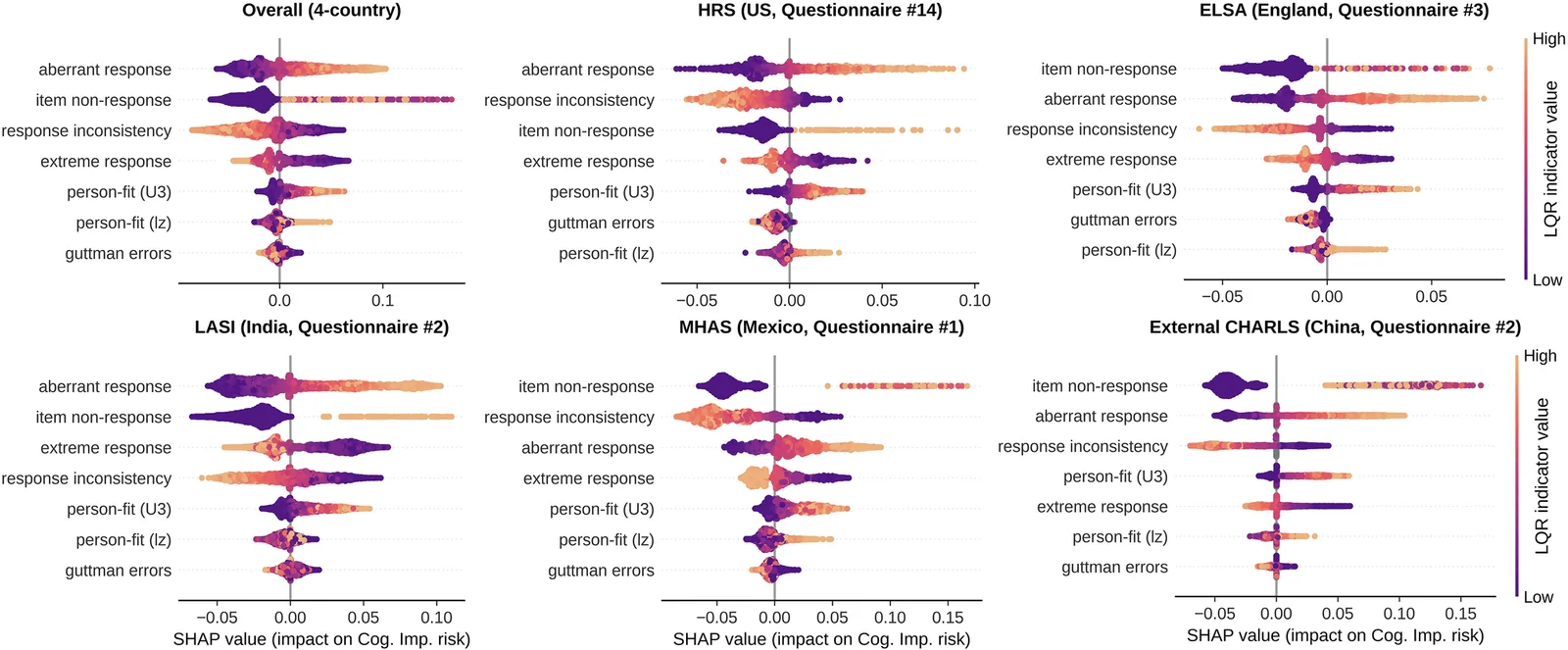
SHAP importance of LQR indicators across countries. Each point represents the SHAP value of a specific low-quality response (LQR) indicator for an individual observation. Indigo and cheese colours represent lower and higher LQR indicator values, respectively. Higher SHAP values (further from zero) indicate a greater impact on the prediction of cognitive impairment risk. SHAP SHapley Additive exPlanation. Cog. Imp. cognitive impairment. Independent test sets (35%): four-country, *N* = 13,376 respondents (56,637 questionnaire-specific observations); external CHARLS, *N* = 2588 respondents (5176 questionnaire-specific observations). CHARLS China Health and Retirement Longitudinal Study, ELSA English Longitudinal Study of Ageing, HRS Health and Retirement Study, LASI Longitudinal Ageing Study in India, MHAS Mexican Health and Ageing Study. Source data are provided as a Source Data file.

Based on the fine-tuned Model II (LQR, demographics, and activity features), we used the best-performing questionnaire from each country to perform SHapley Additive exPlanations (SHAP) analyses, examining the relative contributions of LQR indicators to the prediction of cognitive impairment. Overall, item non-response, aberrant response, and response inconsistency were the most influential indicators cross-nationally (Fig. 3). Aberrant response was the most important indicator in HRS and LASI, whereas item non-response ranked highest in ELSA, MHAS, and external validation on CHARLS. Respondents with higher proportions of unanswered questions and greater deviations from typical response patterns consistently showed increased predicted risk of cognitive impairment, whereas lower response variability was also associated with increased predicted risk.

**Fig. 4:**
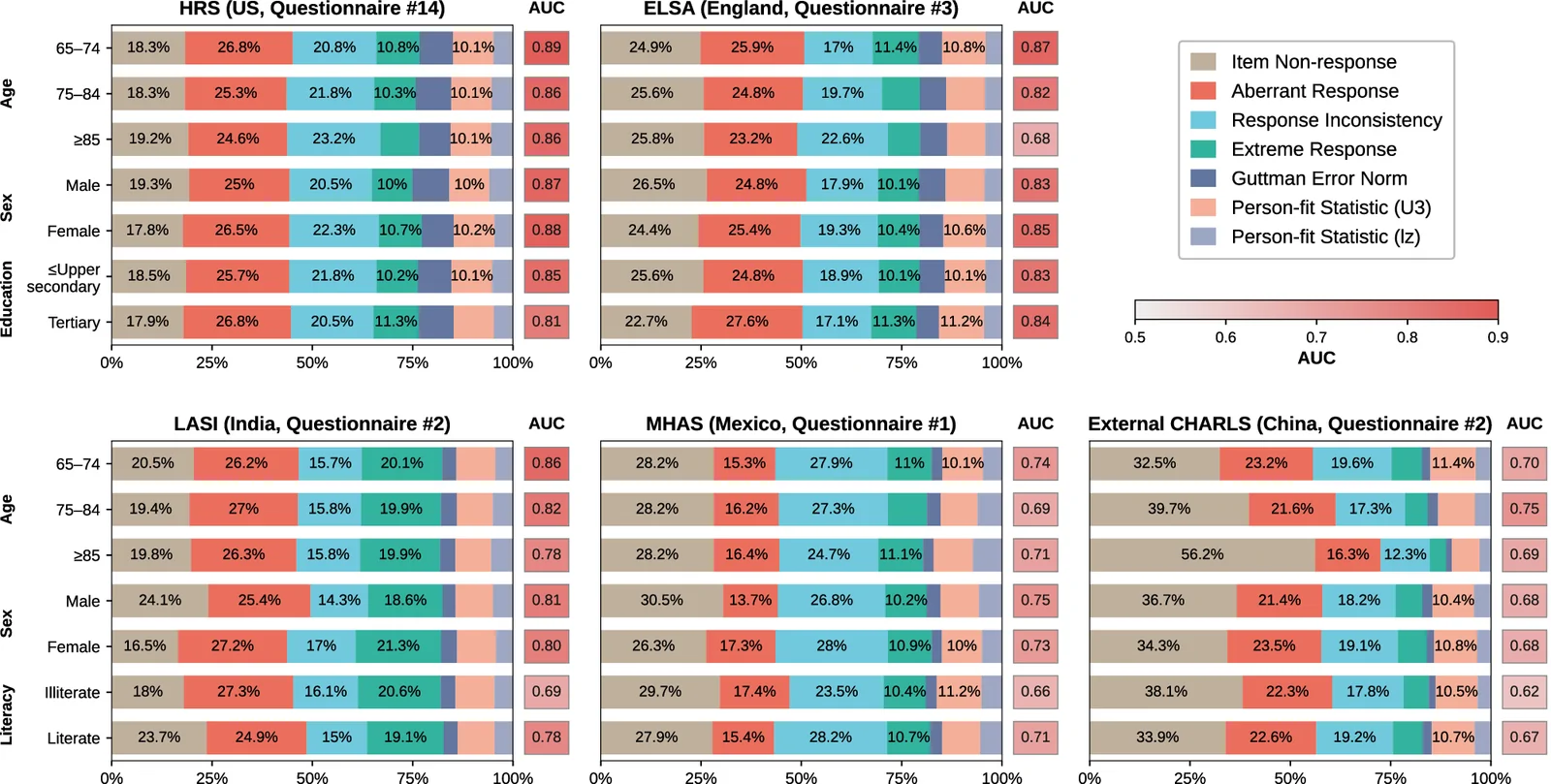
Relative importance of LQR indicators and subgroup-level discrimination performance across countries. The relative contribution of each low-quality response (LQR) indicator was calculated by normalising mean absolute SHAP values within each demographic subgroup. Importance scores ≥ 10% are presented. Colour-coded AUC values represent subgroup discrimination performance across age, sex, education (HRS and ELSA), and literacy (three LMICs: LASI, MHAS, and external validation CHARLS). Independent test sets (35%): four-country, *N* = 13,376 respondents (56,637 questionnaire-specific observations); external CHARLS, *N* = 2588 respondents (5176 questionnaire-specific observations). AUC area under the receiver operating characteristic curve, CHARLS China Health and Retirement Longitudinal Study, ELSA English Longitudinal Study of Ageing, HRS Health and Retirement Study, LASI Longitudinal Ageing Study in India, LMICs low- and middle-income countries, MHAS Mexican Health and Ageing Study, SHAP SHapley Additive exPlanation. Source data are provided as a Source Data file.

The relative importance of LQR indicators remained largely consistent across subgroups defined by age, sex, and education/literacy (Fig. 4), although item non-response contributed more strongly among respondents aged 85 years and older in CHARLS. The model also maintained adequate discrimination across most subgroups, with generally stable performance across age groups and between sexes. However, moderately lower performance was observed among the oldest age group (85+) in ELSA and CHARLS (AUROC of 0.68 and 0.69, respectively), as well as among illiterate respondents in LASI (AUROC of 0.69 for illiterate vs. 0.78 for literate respondents).

Beyond feature-level importance and subgroup consistency, we further examined how questionnaire characteristics shape LQR signals. Questionnaires with stronger predictive performance generally exhibited lower response quality and moderate-to-high text complexity (Supplementary Fig. 3), suggesting that more cognitively demanding questionnaires may amplify informative LQR response behaviours.

### Decision curve analysis

**Fig. 5:**
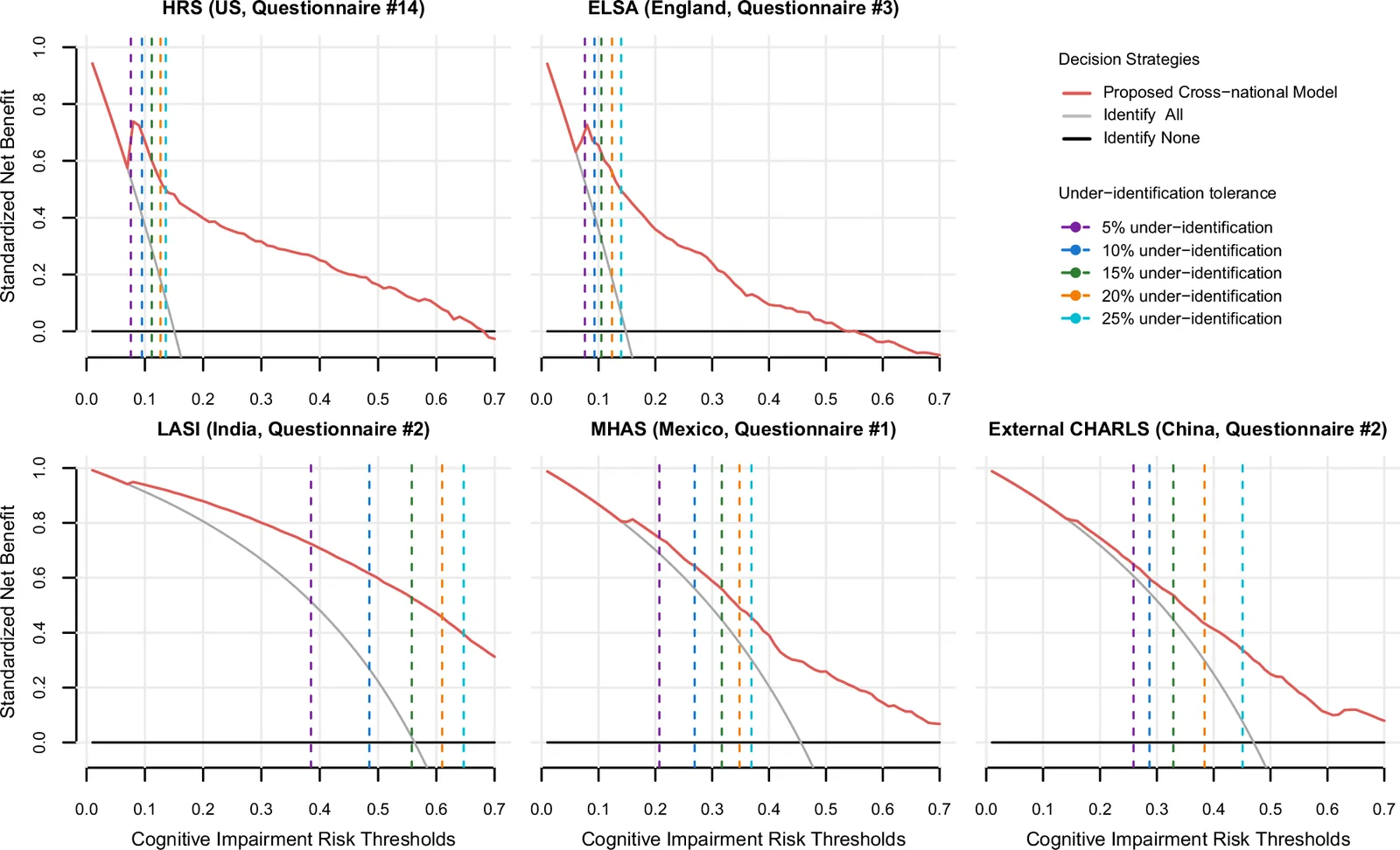
Decision curve analysis for cross-national population-level identification of cognitive impairment risk. Decision curves show the standardised net benefit of the proposed cross-national model across cognitive impairment risk thresholds in HRS, ELSA, LASI, MHAS and external validation CHARLS. The proposed model is compared with two reference strategies: identifying all respondents as high risk and identifying no respondents as high risk. Vertical dashed lines indicate representative thresholds corresponding to 5, 10, 15, 20 and 25% under-identification tolerance. HRS Health and Retirement Study, ELSA English Longitudinal Study of Ageing, LASI Longitudinal Ageing Study in India, MHAS Mexican Health and Ageing Study, CHARLS China Health and Retirement Longitudinal Study. Source data are provided as a Source Data file.

Decision curve analysis showed that our proposed cross-national model for population-level risk identification provided positive standardised net benefit across a wide range of decision thresholds in all five countries, including representative thresholds aligned with varying under-identification tolerance (i.e., 1 − sensitivity), consistently outperforming both extreme reference strategies: classifying all respondents as high risk and classifying none as high risk, which represent the boundaries of decision-making in public health contexts (Fig. 5). These results indicate that the model supports effective population-level identification of cognitive impairment risk across diverse settings by balancing the trade-off between under-identification and over-identification.

## Discussion

Under-recognition of cognitive impairment remains a major global public health challenge, driven in part by reliance on assessments that are not cross-nationally generalisable and resource-intensive—barriers that disproportionately affect LMICs. In this study, we leveraged LQR indicators extracted from large-scale, real-world questionnaire data collected from 45,604 older adults across five countries, including three LMICs, to develop a machine learning pipeline for predicting cognitive impairment. We used easily obtained indicators of questionnaire response behaviours as predictors, and systematically validated the cross-national performance of this pipeline.

Our study demonstrated that cross-national joint training using data from four countries (HRS-US, ELSA-England, LASI-India, MHAS-Mexico) improved predictive performance in both internal and external validation compared to country-specific models. Specifically, the predictive performance as measured by AUROC showed a relative improvement of approximately 14–20% for identifying cognitive impairment in external testing on the culturally distinct CHARLS data from China. Our findings suggest that joint training across multiple countries enabled the model to more effectively capture underlying associative patterns between LQR and cognitive impairment that are shared across culturally diverse populations. These common patterns—later confirmed by our predictor importance analysis—resulted in better performance than training separate models on each country’s data. Besides, in at-risk demographic subgroups (e.g., aged ≥80 years with less than upper secondary education), LQR predictors continued to demonstrate predictive value, indicating that the LQR indicators can be effectively combined with demographic predictors to achieve better performance. This complementary use of both LQR and demographic variables may offer a promising approach for future cross-national applications, enabling more precise risk prediction.

Notably, we observed a phenomenon that we termed cross-national implicit feature transfer, whereby joint cross-national training improved prediction not only in data-rich settings but also in cohorts with fewer available predictors. That is, although activity features like email use and driving status were not measured in the LASI, MHAS, and CHARLS cohorts, incorporating those features from the HRS and ELSA datasets during cross-national joint training still improved predictive performance in all participating cohorts. This improvement persisted even in the CHARLS external validation dataset, where activity features were set to missing (NA) during prediction. Conceptually, this phenomenon parallels mechanisms described in the transfer-learning literature, whereby models trained on heterogeneous datasets learn shared latent representations that capture common underlying patterns^29–31^, and our findings suggest that similar cross-dataset learning can emerge in cross-national joint training with TabPFN. Such a finding is particularly relevant for low- and middle-income settings^3^, where data collection is often constrained, and conventional cognitive assessments may be less feasible. More broadly, it suggests that cross-national modelling approaches may help mitigate disparities in data availability across countries, thereby supporting more equitable predictive performance.

Our analysis of questionnaire complexity revealed that more cognitively demanding questionnaires (i.e., those with higher textual complexity) elicited stronger cross-national LQR signals, enhancing cognitive impairment prediction across countries. This finding is grounded in cognitive load theory, which entails that more complex questions inherently require mobilisation of greater cognitive resources (e.g., working memory, executive function, attentional control), resulting in more sensitive and consistent behavioural indicators of subtle cognitive deficits^20,24,32^. Within individual cohorts (e.g., HRS), the maximum AUROC difference between the most complex and simplest questionnaires reached approximately 0.2 when using the LQR predictors alone. Such a gap underscores the potential for improving the predictive performance through targeted selection or design of complex questionnaires.

Our findings also provide insight into which response behaviours are most informative for prediction. Item non-response, aberrant response, and response inconsistency emerged as the most informative indicators across countries. The contribution patterns of these indicators were broadly consistent across demographic subgroups, supporting their potential as cross-cultural behavioural predictors. At the same time, our results indicate that LQR indicators may partly reflect broader vulnerability factors—such as general frailty and low educational attainment—rather than cognitive impairment alone. This overlap may lead to reduced predictive performance in specific subgroups where such vulnerabilities are more prevalent, including the oldest-old and individuals who are illiterate. Nevertheless, even within these subgroups, model performance (AUROC of 0.68–0.69) remains meaningful and can still support the characterisation of population subgroups at elevated risk, particularly in settings where formal cognitive assessment is not feasible.

The LQR-based approach developed in this study has direct relevance to public health practice and research, with several practical pathways for translation and application. First, as the required predictors are derived from response behaviours rather than questionnaire content, the model could be embedded into routine survey platforms for population-level monitoring, without adding respondent burden. In such settings, model predictions could be used to inform prioritisation of subgroups at elevated risk of cognitive impairment for further cognitive evaluation and other population-based interventions. The decision-curve analysis suggests that our model has value in facilitating efficient population-level identification of cognitive impairment, although its use requires careful consideration of the nuanced trade-off between under-identification and over-identification. Temporarily tolerating higher under-identification rates (i.e., adopting lower sensitivity thresholds) may reduce the short-term assessment or intervention burden, whereas a relatively higher sensitivity threshold may ultimately be preferable. This is because the costs of under-identification may lead to delayed opportunities for intervention, increased long-term economic burden, and widening health inequalities, with particularly severe consequences in resource-constrained low- and middle-income countries (LMICs).

Second, the approach could be applied to existing large-scale, population-based survey datasets that do not currently include direct cognitive assessment, such as the Gallup World Poll^33^, the Understanding Society study^34^, and the European Social Survey^35^. Many of these datasets are publicly available population-based social and economic surveys that face well-recognised challenges in incorporating direct cognitive assessments, including cost, respondent burden, and logistical complexity, rather than a lack of relevance to their substantive research aims^36,37^. The proposed framework enables the enrichment of such datasets with indirect measures of cognitive impairment derived from response behaviours, thereby supporting population-level risk estimation, surveillance, and epidemiological analyses in datasets where formal cognitive assessment is unavailable. This represents a step change in how cognitive health can be studied in public health and epidemiological research, by introducing a data-enrichment approach that repurposes existing survey datasets.

Third, the LQR-based approach could serve as a measurement tool for public health interventions aimed at reducing or preventing the risk of cognitive impairment, complementing traditional cognitive assessment tools. Evaluating such interventions at the population level is often challenging because outcomes may be multidimensional, context-dependent, and difficult to measure consistently over time^38^, particularly in cross-national dementia-prevention trials where harmonising cognitive outcome measures across diverse settings remains an ongoing effort^39^. Its low burden and cross-cultural generalisability make it particularly well suited for population-based interventions, including large-scale trials and routine implementation, especially in contexts targeting diverse populations.

Our study has several limitations. First, the cognitive impairment outcome used in this study was not based on direct clinical diagnosis for the full analytic sample, but was derived from harmonised cognitive factor scores using a multilevel latent class modelling framework anchored on clinically adjudicated cases in HCAP^40,41^. This approach may introduce some uncertainty in outcome classification relative to fully adjudicated clinical diagnoses, and some misclassifications may therefore remain. Second, the study includes three LMICs, but the surveys available in these countries are not as broad as those in HRS and ELSA, which may limit model performance in these settings. Third, although the decision-curve analysis supports potential public health usefulness, it is based on existing observational data rather than first-hand implementation studies and therefore may not fully capture the practical complexity of real-world deployment. Fourth, although TabPFN demonstrated strong predictive performance in this study^29^, it does not explicitly model temporal dependencies and offers more limited interpretability than structured causal models. Fifth, the harmonised cognitive impairment outcome is currently available for only a single wave in each cohort, which precludes full longitudinal modelling. Relatedly, the prospective validation was limited to one earlier wave, corresponding to approximately 2–4 years prior in most cohorts, and the available longitudinal depth varied across cohorts, with LASI contributing only concurrent data from its single available wave. As additional waves of HCAP data and survey data accumulate across cohorts, future research should extend the prospective prediction interval, incorporate longitudinal modelling, and explore approaches better suited to temporal and causal structure.

In summary, these findings suggest that routinely captured questionnaire response behaviours can provide a practical and scalable signal for early identification and surveillance of cognitive impairment risk across culturally diverse populations. By leveraging information already embedded within existing surveys, LQR-based prediction offers a low-burden approach that complements formal cognitive assessment and may support population-level surveillance and evidence generation for future evaluation, particularly in diverse and resource-constrained settings where conventional cognitive assessment methods remain difficult to deliver at scale.

## Methods

### Ethics statement

This study complies with all relevant ethical regulations. Ethical approval for the original cohort data collection was obtained from the following institutions: HRS (approved by the University of Michigan Institutional Review Board); ELSA (approved by the South Central-Berkshire Research Ethics Committee, 13/SC/0532 and 15/SC/0526); LASI (approved by the Indian Council of Medical Research, Delhi, and the Institutional Review Board of the International Institute for Population Sciences, Mumbai); MHAS (approved by the institutional review boards of the University of Texas Medical Branch and the Instituto Nacional de Estadística y Geografía, Mexico); and CHARLS (approved by the Biomedical Ethics Review Committee of Peking University, IRB00001052-11015). All participants in the original cohort studies provided written informed consent. This study involves secondary analysis of de-identified, controlled-access data and was approved by the University of Surrey Research Integrity and Governance Office (FHMS 21-22 216 EGA) and the University of Southern California Institutional Review Board (UP-22-00147).

### Study population

We used data from five population-based ageing cohort studies (HRS [US], ELSA [England], LASI [India], MHAS [Mexico], CHARLS [China])^42–46^, reflecting diverse cultural, socioeconomic, and policy contexts, each incorporating validated psychosocial questionnaires suitable for generating LQR indicators^25^. Crucially, these studies implemented the Harmonised Cognitive Assessment Protocol (HCAP) sub-studies^40,47–50^, enabling standardised assessment of cognitive impairment through harmonised cognitive factor scores^51^. These factor scores capture cognitive abilities across multiple domains, derived using consistent psychometric models to reduce measurement bias due to language or cultural differences.

In total, 45,604 participants covering 29 psychosocial questionnaires overall were included (Supplementary Table 1 and Table 2). Participants were eligible if they: (1) completed the psychosocial questionnaires from the core or harmonised surveys during each country’s corresponding HCAP wave (HRS Wave 13 [2016], ELSA Wave 7 [2014-15] and Wave 8 [2016-17], LASI Wave 1 [2017-19], MHAS Wave 4 [2015], CHARLS Wave 4 [2018]); (2) were aged ≥65 years at the time of the HCAP wave; and (3) participated via non-proxy interviews.

Participant demographics, including age and self-reported sex, are summarised in Supplementary Table 1. Sex was determined by self-report in the original cohort surveys and was included as a prespecified demographic predictor in the models and in sex-based subgroup analyses. Participant compensation for the original surveys was managed by the respective cohort study teams; no compensation was provided for this secondary analysis.

This study is reported in accordance with the TRIPOD + AI (Transparent Reporting of a Multivariable Prediction Model for Individual Prognosis or Diagnosis + Artificial Intelligence) guidelines^52^, with the completed checklist provided in Supplementary Table 8.

### Definition of cognitive impairment status

In this study, we utilised cross-nationally and statistically harmonised cognitive scores (available from Gross et al.^51^), encompassing general cognitive performance and four specific domains—memory, executive function, language, and orientation—to derive cognitive impairment status. Because these harmonised scores were available only for participants in the Harmonised Cognitive Assessment Protocol (HCAP) sub-studies of HRS, ELSA, CHARLS, LASI, and MHAS, we first employed a semi-supervised learning approach to predict cognitive assessment scores for the full cohort samples^53^. This approach demonstrated high predictive accuracy, as evidenced by its performance on an independently reserved testing set: e.g., for general cognitive performance, the Root Mean Square Error (RMSE) ranged from 0.38 (CHARLS, China) to 0.44 (LASI, India), *R*² ranged from 0.83 (MHAS, Mexico) to 0.85 (ELSA, England), and Concordance Correlation Coefficient (CCC) ranged from 0.85 (LASI, India) to 0.93 (ELSA, England).

In population-based studies where cognitive assessment scores are available, cognitive impairment has traditionally been classified using statistical thresholds. A common approach defines dementia as scoring at least 1.5 standard deviations (SD) below the age-adjusted—or sometimes age- and education-adjusted—norm, and MCI or cognitive impairment no dementia (CIND) as scoring 1.0–1.5 SD below the norm^54,55^. However, applying this approach to our multi-country datasets yielded implausibly low estimates of cognitive impairment in groups where the risk of cognitive impairment is known to be high. For example, in India, China, and Mexico, relatively older age groups exhibited lower rates of impairment than younger groups, a pattern inconsistent with epidemiological evidence^56^. This anomaly arises because the very low levels of education and high rates of illiteracy in these groups result in uniformly low cognitive scores with restricted variability. Consequently, the SD is compressed, and few individuals fall 1 or 1.5 SD below the adjusted mean.

To address these limitations, we adopted a Bayesian multilevel latent class modelling approach to derive cognitive impairment status directly from the joint distribution of the harmonised cognitive scores. Rather than imposing externally defined cut-offs, this approach probabilistically identifies latent subgroups characterised by progressively lower levels of multivariate cognitive performance, corresponding to cognitively normal, MCI/CIND, and dementia. Class membership is estimated based on individuals’ profiles across general cognition and the four cognitive domains, allowing cognitive status to be inferred as a latent construct rather than determined by a single thresholded score. This probabilistic formulation also accommodates uncertainty in classification, which is particularly relevant in population-based samples where cognitive performance varies continuously.

The latent class model was implemented within a multilevel framework that jointly estimated data from all participating countries. This structure places individuals from different national contexts on a common latent scale, thereby facilitating cross-national comparability, while allowing the associations between cognitive scores and latent class membership to vary across countries. Such flexibility is essential in cross-national settings marked by substantial heterogeneity in educational attainment, literacy, and test performance distributions, where uniform SD-based thresholds are shown to perform poorly. By allowing class boundaries to emerge empirically and to adapt across populations, the latent class approach avoids imposing fixed cut-offs that may be inappropriate in specific country contexts.

To enhance clinical interpretability and stabilise estimation, the model incorporated anchor cases from HCAP sub-studies of HRS and LASI in which cognitive status had been clinically adjudicated^40,41^. For these individuals, class membership was treated as known during model estimation, providing reference points that guided the alignment and interpretation of latent classes across countries, while leaving class membership for most participants to be estimated probabilistically. Cognitive status was derived in two sequential stages: first, distinguishing cognitively normal individuals from those with any cognitive impairment, and subsequently differentiating dementia from MCI among those identified as impaired. This staged modelling strategy reflects the hierarchical nature of cognitive impairment and improves discrimination between adjacent categories.

The resulting classifications demonstrated their face validity. They exhibited expected monotonic associations with established risk factors for cognitive impairment, including increasing age and lower educational attainment, and avoided the counterintuitive age gradients observed when conventional SD-based thresholds were applied to the same harmonised cognitive scores (Supplementary Table 9). The combination of harmonised cognitive measurement, semi-supervised prediction of scores for full-cohort samples, and multilevel latent class modelling provides a flexible, data-driven, and clinically anchored framework for identifying cognitive impairment in cross-national population studies.

### Construction of LQR feature set

We generated seven LQR indicators from each multi-item questionnaire in the five cohorts. In total, there were 29 different questionnaires, each consisting of four to seven brief items rated on 3–7 point Likert scales (Supplementary Tables 2, 3). LQR indicators were primarily computed within an item response theory (IRT) framework to quantify deviations from expected response patterns^21,25,57^. These include: (1) item non-response—proportion of skipped items; (2) extreme response—tendency to choose extreme options (e.g., always or never), estimated using a two-dimensional nominal response model that separates extremity in responding from the underlying trait level^58^; (3) response inconsistency—log of within-person residual variability in item responses relative to a fitted factor model (e.g., randomness or instability); (4) Aberrant response pattern—M-distance statistic (p-value transformed), measuring deviation from the cohort’s modal response pattern^59^; (5) Guttman errors– violations of expected logical order in ordinal or hierarchical item^60^; and (6–7) Polytomous person-fit statistics – lz, which quantifies overall misfit of an individual’s response pattern under the IRT model, and U3, which captures aberrance based on the accumulation and structure of Guttman errors^61^.

We then constructed two predictor sets. Group 1 included the seven LQR indicators, survey language (Supplementary Table 4), and three easily obtainable demographic variables (age, sex, and education level). Group 2 additionally incorporated four daily activity variables available only in HRS and ELSA to demonstrate the cross-national implicit feature transfer phenomenon by examining whether combining these variables with LQR indicators could enhance cross-national predictive performance, particularly for LMIC cohorts in which these variables were not collected (Supplementary Table 6).

### TabPFN model and fine-tuning experiments

We adopted the Tabular Prior-Data Fitted Network (TabPFN) with few-shot learning capabilities as the core model for supervised learning^29^. TabPFN is a Transformer-based tabular foundation model trained on diverse synthetic and real-world tabular datasets, designed to approximate the Bayesian posterior predictive distribution (PPD) and enable rapid in-context learning (ICL). Given an observed dataset ${{{\mathcal{D}}}}$, TabPFN estimates the posterior predictive distribution of the target variable y given input features x as^62^:

1 $$p\left(y | x,{{{\mathcal{D}}}}\right)=\int _{\Pi }\,\pi \left(y | x\right)p\left(\pi | {{{\mathcal{D}}}}\right)d\pi$$

where Π denotes the space of all possible joint distributions, π represents a specific joint distribution, $p\left(\pi,|,{{{\mathcal{D}}}}\right)$ is the posterior over π given ${{{\mathcal{D}}}}$.

Unlike conventional supervised training methods, TabPFN’s ICL stage does not require additional gradient updates or hyperparameter tuning, directly generating prediction probabilities through a single forward pass (this stage still requires a small support set of training samples to construct contextual information, hereafter referred to as ICL training)^29^. TabPFN also inherently handles missing data and captures implicit dependencies among variables via conditional modelling.

To adapt TabPFN’s pretrained weights to better capture LQR-specific response behaviour patterns, we performed full fine-tuning using only the LQR predictors (seven LQR indicators and survey language). Inspired by the TabPFN developers and the principle of curriculum learning (i.e., gradually increasing task complexity to achieve better predictive performance)^63,64^, we designed a two-stage strategy: first, fine-tuning the default TabPFN weights on a binary classification task (cognitively normal vs cognitive impairment) with clearer decision boundaries, then transferring the binary-task weights to the three-class classification task (normal vs MCI vs dementia) for further fine-tuning to obtain the final weights. The fine-tuned weights still required ICL training with the full predictor sets (Groups 1 and 2). Both fine-tuning stages employed the cross-entropy loss function for model optimisation:

2 $${L}_{{CE}}^{{{{\rm{binary}}}}\,}\left(y,\hat{y}\right)=-{\sum}_{i}\,\left[{y}_{i}\log \left({\hat{y}}_{i}\right)+\left(1-{y}_{i}\right)\log \left(1-{\hat{y}}_{i}\right)\right]$$

3 $${L}_{{CE}}^{{{{\rm{multiclass}}}}\,}\left(y,\hat{y}\right)=-{\sum}_{i}\,{\sum}_{c}\,{y}_{i,c}\log \left({\hat{y}}_{i,c}\right)$$

During fine-tuning, we used mixed-precision training and gradient scaling for computational efficiency^65^, gradient clipping to prevent gradient explosion^66^, and gradient accumulation to simulate larger batch sizes under limited resources^67^. AdamW optimiser with schedule-free learning rate scheduling and adaptive early stopping was employed to mitigate overfitting and improve generalisation^68^.

### Cross-national joint training and evaluation strategy

We developed a cross-national predictive model using the TabPFN classifier to identify cognitive impairment, and compared it against country-specific models trained separately on data from individual countries. The dataset was reshaped into long format, treating each participant’s questionnaire-specific LQR indicators as independent observations. Missing LQR indicators were not imputed to preserve authentic response patterns (e.g., item non-response patterns), which are themselves indicative of subtle cognitive deficits^21,22,25^. The final stacked dataset structure and missingness of LQR indicators are described in Supplementary Table 10.

To assess the statistical suitability of cross-national joint training, we conducted a variance decomposition analysis using a linear mixed-effects model to estimate random-effect variance components for LQR indicators at the country, individual, and indicator levels^69^. The resulting intraclass correlation coefficients (ICC) indicated that^70^: (1) the country-level variance was 0.077 (ICC = 3.65%), suggesting limited cross-national variability; (2) the within-country individual-level variance was 0.014 (ICC = 0.68%), indicating minimal inter-individual variability; and (3) the residual variance at the within-individual indicator level was 2.018 (ICC = 95.67%), suggesting that most variability arose from differences between indicators within the same individual. The country-level ICC was well below the conventional 5% threshold, supporting the statistical justification for treating each participant’s questionnaire-specific LQR indicators as independent observations in the joint training strategy.

Throughout the entire model analysis, we employed stratified random sampling by questionnaire and cognitive status, ensuring each participant was assigned to a single data subset and preserving the original distribution of cognitive status. Data from HRS, ELSA, MHAS, and LASI were used for joint training, while CHARLS—with its distinct cultural and economic background—served as a fully independent external validation set to rigorously assess cross-national generalisation. The joint training dataset was partitioned as follows: 10% for the ICL training set (reflecting recommended practices for TabPFN context window construction and simulating low-resource learning scenarios)^29^; 30% for fine-tuning (only the LQR predictors; split into 80% fine-tuning training and 20% fine-tuning validation); 25% for validation (model calibration and generalisation consistency checks); and 35% as the independent test set (relatively large to enhance robustness and reliability under minority-class sparsity)^71^. The CHARLS dataset was split using the same proportions, with 35% for testing and 10% for ICL training. Data-splitting details are provided in Supplementary Table 10.

We evaluated the predictive performance at three analytical levels. (1) Questionnaire level: Calculated one-vs-rest AUROC metrics for cognitively normal vs cognitive impairment (MCI and dementia), MCI vs others, dementia vs others, and the macro AUROC (mean of the three metrics), with 95% confidence intervals derived via bootstrapping. (2) Within-country level: Calculated mean, SD, maximum, and minimum AUROC values across all questionnaires within each country. (3) Cross-country level: Calculated mean, SD, maximum, and minimum AUROC values across all questionnaires from the four jointly-trained countries.

### Prospective validation

Prospective validation analyses were conducted using LQR indicators derived from questionnaire responses collected approximately 2 years earlier (ELSA: wave 6 to waves 7–8; CHARLS: waves 3–4; MHAS: waves 3–4), and approximately 4 years earlier for HRS (wave 11 to wave 13) due to its alternating panel design. This design was chosen to maximise retention of the four-country joint-training framework while using the earliest available prior-wave LQR indicators for each cohort.

### Sensitivity analyses

We conducted sensitivity analyses using 100 random-seed combinations to assess the robustness and stability of predictive results. TabPFN performance was further compared with three traditional ensemble machine learning methods, including XGBoost^72^, LightGBM^73^, and CatBoost^74^ all with default parameters, across varying training data proportions (10–100% in 10% increments). We also investigated the impact of different fine-tuning set sizes (25, 50, 75, 100%) on classification performance.

### Questionnaire complexity and response quality analyses

We examined whether text complexity and overall response quality were associated with predictive performance. As some common readability metrics (e.g., Flesch–Kincaid Grade Level) are language-specific, we instead quantified complexity using three cross-lingual measures applicable across the representative questionnaire languages (HRS and ELSA: English; LASI: Hindi; MHAS: Spanish; CHARLS: Chinese). These measures were: (1) Semantic comprehension difficulty, estimated using the cross-lingual pre-trained model XLM-RoBERTa via masked-language modelling loss^75^; (2) Semantic rarity, calculated via cosine similarity in embedding space to indicate the abstraction or distinctiveness of item content; and (3) Difficult word ratio, computed using the cross-lingual word frequency database (wordfreq) to capture lexical complexity^76^. For each questionnaire, these complexity measures were summed, standardised across countries (Z-score), and transformed into percentile ranks (0–100%), reflecting the overall relative complexity distribution.

Response quality was summarised per respondent as the mean of seven z-scored LQR indicators (LQR composite score), aligned so that higher scores indicated better quality. Individual composite scores were then converted into percentile ranks (0–100%), and these percentiles were averaged within each questionnaire to reflect questionnaire-level response quality.

### Model interpretation and decision curve analysis

For interpretability, we selected the best-performing questionnaire from each country based on the final fine-tuned model incorporating LQR indicators, demographic variables, and activity features (where available). Predicted three-class probabilities (cognitively normal, MCI, dementia) were aggregated into a binary outcome (cognitively normal vs. cognitive impairment) to align with common screening and referral decisions in population health contexts. We applied SHapley Additive exPlanations (SHAP)^77^, a game-theoretic approach that decomposes individual predictions into additive feature-level contributions. SHAP values were computed for each respondent and summarised as mean absolute values to quantify the relative importance of each LQR indicator both cross-nationally and within each country. To examine robustness of interpretation, we further assessed consistency of LQR indicator contributions across key demographic subgroups by calculating normalised mean absolute SHAP values within strata, using age, sex, and education in HRS and ELSA, and age, sex, and literacy in LASI, MHAS, and CHARLS.

To assess the potential public health value of the model beyond discrimination metrics, we conducted decision curve analysis (DCA)^78^. DCA evaluates the net benefit of using a prediction model across a range of decision thresholds by explicitly weighing the relative consequences of true positives (correctly identifying high-risk individuals) against false positives (incorrectly identifying low-risk individuals as high-risk). Unlike accuracy-based metrics such as AUROC, which are agnostic to downstream decisions, DCA is specifically designed to inform whether a model may improve decision-making compared with simple reference strategies, such as identifying all individuals as high risk or identifying none.

In this study, DCA was used to simulate population-level identification strategies for cognitive impairment risk under varying tolerances for under-identification (i.e., 1 − sensitivity). Decision thresholds, therefore, reflect different policy or implementation scenarios, ranging from conservative approaches prioritising specificity to more inclusive strategies prioritising early detection. Predicted probabilities were calibrated using Platt scaling prior to DCA to ensure that estimated risks corresponded to observed outcome frequencies^79^, a necessary condition for meaningful decision analysis (Supplementary Fig. 4). Net benefit was standardised to facilitate comparison across countries with differing base rates of cognitive impairment.

This framework allows evaluation of whether the proposed model could support efficient population-level identification of individuals at elevated cognitive impairment risk across diverse national contexts, without assuming a single optimal threshold. By explicitly representing the trade-off between over-identification and under-identification, DCA provides an interpretable link between model predictions and real-world public health decision-making, particularly in settings where resources for follow-up cognitive assessment are limited, and prioritisation is necessary.

### Computational hardware and software

Computational tasks related to model development were performed on a cloud-based interactive computing platform equipped with an Intel Xeon CPU (2.20 GHz, 12 cores) and one NVIDIA A100 GPU (40GB, CUDA 12.4), using Python versions 3.12.11 (main analyses) and 3.11.13 (fine-tuning experiments). The LQR indicators were generated using SAS for Windows (version 9.4), with embedded execution of R (version 4.2.1) and Mplus (version 7.11) code. Key Python packages used include TabPFN (v2.0.9), XGBoost (v2.1.4), LightGBM (v4.5.0), CatBoost (v1.2.8), SHAP (v0.48.0), NumPy (v2.0.2), pandas (v2.2.2), PyTorch (v2.6.0), scikit-learn (v1.5.2), scipy (v1.15.3), matplotlib (v3.10.0), and seaborn (v0.12.2). Full package dependencies are listed in the GitHub repository listed in the Code Availability section.

### Statistics & reproducibility

No statistical method was used to predetermine sample size; the study used existing data from five population-based ageing cohort studies. The sample comprised all eligible participants aged 65 years or older who completed the psychosocial questionnaires during the relevant survey wave and participated via non-proxy interviews. Participants who did not meet these criteria were excluded, as described in the Study population section. The study was observational, and the experiments were not randomised. The investigators were not blinded to allocation during experiments and outcome assessment because no intervention, treatment allocation or experimental group assignment was involved. Model development and evaluation used prespecified train, validation and test splits, with an independent external validation cohort from CHARLS. Sensitivity analyses were conducted across repeated random splits and reduced training-set proportions to assess reproducibility and robustness.

### Reporting summary

Further information on research design is available in the Nature Portfolio Reporting Summary linked to this article.

## Supplementary information


Supplementary Information
Reporting Summary
Transparent Peer Review file


## Source data


Source Data


## Data Availability

The datasets analysed in this study are from five ageing cohorts accessed through registration or application procedures: the Health and Retirement Study (HRS), the English Longitudinal Study of Ageing (ELSA), the Longitudinal Aging Study in India (LASI), the Mexican Health and Aging Study (MHAS), and the China Health and Retirement Longitudinal Study (CHARLS), including relevant Harmonised Cognitive Assessment Protocol (HCAP) data. These cohort data are not owned by the authors and cannot be redistributed by the authors. They are available under controlled access through the Gateway to Global Aging Data (https://g2aging.org/) and the relevant cohort data portals, subject to registration, application where required, and the applicable data-use agreements. Access requests are handled by the relevant data providers according to their own access procedures, eligibility criteria, response timelines and data-use terms. Participant-level derived low-quality response indicators are based on these controlled-access cohort questionnaire data and are therefore subject to the original cohort data-use agreements rather than deposited separately. Source data are provided with this paper.

## Code availability

The analysis code and fine-tuned model weights used in this study are available via GitHub at https://github.com/ocean8800v/cross-national-cognitive-ai and archived on Zenodo at https://doi.org/10.5281/zenodo.20651481^80^.

## References

[1] World Health Organization. Dementia. https://www.who.int/health-topics/dementia (WHO, 2025).

[2] Alzheimer’s Disease International. Dementia statistics. https://www.alzint.org/about/dementia-facts-figures/dementia-statistics/ (ALZINT, 2025).

[3] WHO. A blueprint for dementia research. *Geneva**:**World Health Organization* Licence: CC BY-NC-SA 3.0 IGO (WHO, 2022).

[4] Alzheimer’s disease facts and figures. *Alzheimer’s Dementia* **20**, 3708–3821 (2024). 10.1002/alz.13809 PMC11095490 38689398

[5] van Dyck, C. H. et al. Lecanemab in early Alzheimer’s disease. *N. Engl. J. Med.* **388**, 9–21 (2023). 36449413 10.1056/NEJMoa2212948

[6] Sims, J. R. et al. Donanemab in early symptomatic Alzheimer's disease: the TRAILBLAZER-ALZ 2 randomized clinical trial. *JAMA* **330**, 512–527 (2023). 37459141 10.1001/jama.2023.13239 PMC10352931

[7] Devenney, E. & Hodges, J. R. The mini-mental state examination: pitfalls and limitations. *Pract. Neurol.* **17**, 79–80 (2017). 27903765 10.1136/practneurol-2016-001520

[8] Nasreddine, Z. S. MoCA test mandatory training and certification: what is the purpose? *J. Am. Geriatrics Soc.* **68**, 444–445 (2020). 10.1111/jgs.16267 31792923

[9] Borchert, R. J. et al. Artificial intelligence for diagnostic and prognostic neuroimaging in dementia: a systematic review. *Alzheimer's Dement* **19**, 5885–5904 (2023). 37563912 10.1002/alz.13412

[10] Winchester, L. M. et al. Artificial intelligence for biomarker discovery in Alzheimer’s disease and dementia. *Alzheimer's Dement* **19**, 5860–5871 (2023). 37654029 10.1002/alz.13390 PMC10840606

[11] Yokomizo, J. E., Simon, S. S. & Bottino, C. M. de C. Cognitive screening for dementia in primary care: a systematic review. *Int. Psychogeriatr.* **26**, 1783–1804 (2014). 25023857 10.1017/S1041610214001082

[12] Pellicer-Espinosa, I. & Díaz-Orueta, U. Cognitive screening instruments for older adults with low educational and literacy levels: a systematic review. *J. Appl Gerontol.* **41**, 1222–1231 (2022). 34856843 10.1177/07334648211056230 PMC8966106

[13] Wu, M. et al. Validity and usability for digital cognitive assessment tools to screen for mild cognitive impairment: a randomized crossover trial. *J. Neuroeng. Rehabilit.* **22**, 132 (2025). 10.1186/s12984-025-01665-1 PMC12153127 40500744

[14] O’Driscoll, C. & Shaikh, M. Cross-cultural applicability of the montreal cognitive assessment (MoCA): a systematic review. *J. Alzheimers Dis.* **58**, 789–801 (2017). 28482634 10.3233/JAD-161042

[15] National Institutes of Health. Cognitive Assessment Considerations: Understanding the Evidence. https://www.nia.nih.gov/health/cognitive-assessment-considerations-understanding-evidence (NIH, 2023).

[16] Singh, S. G., Das, D., Barman, U. & Saikia, M. J. Early Alzheimer’s disease detection: a review of machine learning techniques for forecasting transition from mild cognitive impairment. *Diagnostics* **14**, 1759 (2024). 39202248 10.3390/diagnostics14161759 PMC11353639

[17] Zang, G. Y., Rao, K., Wu, A. T., Tang, Y. & Zhang, Z. Cognitive impairment screening strategy to reduce the burden of Alzheimer’s disease in Shanghai: a system dynamics approach. *J. Alzheimer's Dis. Rep.* **9**, 25424823251337941 (2025). 40330107 10.1177/25424823251337941 PMC12053055

[18] Bond, J. et al. Screening for cognitive impairment, Alzheimer’s disease and other dementias: Opinions of European caregivers, payors, physicians and the general public. * J. Nutr., health aging* **14**, 558–562 (2010). 20818471 10.1007/s12603-010-0268-6 PMC12880248

[19] Sabbagh, M. N. et al. Rationale for early diagnosis of mild cognitive impairment (MCI) supported by emerging digital technologies. * J. Prev. Alzheimer's. Dis.* **7**, 158–164 (2020). 32463068 10.14283/jpad.2020.19

[20] Tourangeau, R., Rips, L. J. & Rasinski, K. *The Psychology of Survey Response*. 10.1017/CBO9780511819322.(Cambridge University Press, 2000).

[21] Schneider, S. et al. Subtle mistakes in self-report surveys predict future transition to dementia. *Alzheimer's Dement* **13**, e12252 (2021). 10.1002/dad2.12252 PMC8652408 34934800

[22] Schneider, S. et al. Quality of survey responses at older ages predicts cognitive decline and mortality risk. *Innov. Aging* **6**, igac027 (2022). 35663275 10.1093/geroni/igac027 PMC9155162

[23] Jin, H., Junghaenel, D. U., Orriens, B., Lee, P.-J. & Schneider, S. Developing early markers of cognitive decline and dementia derived from survey response behaviors: protocol for analyses of preexisting large-scale longitudinal data. *JMIR Res. Protoc.* **12**, e44627 (2023). 36809337 10.2196/44627 PMC9993229

[24] Schneider, S. et al. Can you tell people’s cognitive ability level from their response patterns in questionnaires? *Behav. Res.* 10.3758/s13428-024-02388-2. (2024). PMC11362444 38528247

[25] Schneider, S. et al. Cognitive functioning and the quality of survey responses: an individual participant data meta-analysis of 10 epidemiological studies of aging. *J. Gerontol. B Psychol. Sci. Soc. Sci.* **79**, gbae030 (2024). 38460115 10.1093/geronb/gbae030 PMC10998342

[26] Gao, H. et al. Early identification of cognitive impairment in community environments through modeling subtle inconsistencies in questionnaire responses: machine learning model development and validation. *JMIR Formative Res.* **8**, e54335 (2024). 10.2196/54335 PMC11602764 39536306

[27] Gateway to Global Aging Data. *National Institute on Aging* https://www.nia.nih.gov/research/resource/gateway-global-aging-data.

[28] Vermunt, J. K. Multilevel latent class models. *Sociol. Methodol.* **33**, 213–239 (2003).

[29] Hollmann, N. et al. Accurate predictions on small data with a tabular foundation model. *Nature* **637**, 319–326 (2025). 39780007 10.1038/s41586-024-08328-6 PMC11711098

[30] Ye, H.-J., Liu, S.-Y. & Chao, W.-L. A closer look at TabPFN v2: understanding its strengths and extending its capabilities. In* Advances in Neural Information Processing Systems* 38, 135605–135637 (Curran Associates, Inc., 2025).

[31] Islam, S. et al. A comprehensive survey on applications of transformers for deep learning tasks. *Expert Syst. Appl.* **241**, 122666 (2024).

[32] Knäuper, B., Belli, R. F., Hill, D. H. & Herzog, A. R. Question difficulty and respondents’ cognitive ability: the effect on data quality. *J. Off. Stat.* **13**, 181 (1997).

[33] Tortora, R. D., Srinivasan, R. & Esipova, N. The Gallup World Poll. in *Survey Methods in Multinational, Multiregional, and Multicultural Contexts* 535–543 10.1002/9780470609927.ch31.(Wiley, 2010).

[34] Buck, N. & McFall, S. Understanding society: design overview. *Longitud. Life Course Stud.* **3**, 5–17 (2012).

[35] Davidov, E., Schmidt, P. & Schwartz, S. H. Bringing values back in: the adequacy of the European social survey to measure values in 20 countries. *Public Opin. Q* **72**, 420–445 (2008).

[36] Hofer, S. M. & Alwin, D. F. *Handbook of Cognitive Aging: Interdisciplinary Perspectives*. (SAGE, 2008).

[37] Weir, D. R. et al. Reducing case ascertainment costs in U.S. population studies of Alzheimer’s disease, dementia, and cognitive impairment—Part 1. *Alzheimer’s. Dement.* **7**, 94–109 (2011). 21255747 10.1016/j.jalz.2010.11.004 PMC3044596

[38] Datta, J. & Petticrew, M. Challenges to evaluating complex interventions: a content analysis of published papers. *BMC Public Health* **13**, 568 (2013). 23758638 10.1186/1471-2458-13-568 PMC3699389

[39] Kivipelto, M. et al. Worldwide fingers network: a global approach to risk reduction and prevention of dementia. *Alzheimer's Dement* **16**, 1078–1094 (2020). 32627328 10.1002/alz.12123 PMC9527644

[40] Manly, J. J. et al. Estimating the prevalence of dementia and mild cognitive impairment in the US: the 2016 health and retirement study harmonized cognitive assessment protocol project. *JAMA Neurol.* **79**, 1242–1249 (2022). 36279130 10.1001/jamaneurol.2022.3543 PMC9593315

[41] Lee, J. et al. Prevalence of dementia in India: national and state estimates from a nationwide study. *Alzheimer's Dement* **19**, 2898–2912 (2023). 36637034 10.1002/alz.12928 PMC10338640

[42] Sonnega, A. et al. Cohort profile: the Health and Retirement Study (HRS). *Int J. Epidemiol.* **43**, 576–585 (2014). 24671021 10.1093/ije/dyu067 PMC3997380

[43] Steptoe, A., Breeze, E., Banks, J. & Nazroo, J. Cohort profile: the English Longitudinal Study of Ageing. *Int J. Epidemiol.* **42**, 1640–1648 (2013). 23143611 10.1093/ije/dys168 PMC3900867

[44] Perianayagam, A. et al. Cohort profile: the longitudinal aging study in India (LASI). *Int J. Epidemiol.* **51**, e167–e176 (2022). 35021187 10.1093/ije/dyab266 PMC9365624

[45] Wong, R., Michaels-Obregon, A. & Palloni, A. Cohort profile: the Mexican health and aging study (MHAS). *Int J. Epidemiol.* **46**, e2–e2 (2017). 25626437 10.1093/ije/dyu263 PMC5837398

[46] Zhao, Y., Hu, Y., Smith, J. P., Strauss, J. & Yang, G. Cohort profile: the China Health and Retirement Longitudinal Study (CHARLS). *Int J. Epidemiol.* **43**, 61–68 (2014). 23243115 10.1093/ije/dys203 PMC3937970

[47] Cadar, D. et al. Cohort profile update: the Harmonised Cognitive Assessment Protocol Sub-study of the English Longitudinal Study of Ageing (ELSA-HCAP). *Int. J. Epidemiol.* **50**, 725–726i (2021). 33370436 10.1093/ije/dyaa227 PMC8271185

[48] Lee, J. & Dey, A. B. Introduction to LASI-DAD: the Longitudinal Aging Study in India-Diagnostic Assessment of Dementia. *J. Am. Geriatr. Soc.* **68**, S3–S4 (2020). 32815600 10.1111/jgs.16740 PMC7513796

[49] Mejia-Arango, S. et al. The Mexican cognitive aging ancillary study (Mex-Cog): study design and methods. *Arch. Gerontol. Geriatr.* **91**, 104210 (2020). 32781379 10.1016/j.archger.2020.104210 PMC7854788

[50] Meng, Q. et al. Validation of neuropsychological tests for the China Health and Retirement Longitudinal Study Harmonized Cognitive Assessment Protocol. *Int Psychogeriatr.* **31**, 1709–1719 (2019). 31309907 10.1017/S1041610219000693 PMC8082093

[51] Gross, A. L. et al. Harmonisation of later-life cognitive function across national contexts: results from the Harmonized Cognitive Assessment Protocols. * Lancet Healthy Longev.* **4**, e573–e583 (2023). 37804847 10.1016/S2666-7568(23)00170-8 PMC10637129

[52] Collins, G. S. et al. TRIPOD + AI statement: updated guidance for reporting clinical prediction models that use regression or machine learning methods. *BMJ* **385**, e078378 (2024). 38626948 10.1136/bmj-2023-078378 PMC11019967

[53] Jin, H., Crimmins, E., Langa, K. M., Dey, A. B. & Lee, J. Estimating the prevalence of dementia in India using a semi-supervised machine learning approach. *Neuroepidemiology* **57**, 43–50 (2023). 36617419 10.1159/000528904 PMC10038923

[54] Chertkow, H., Feldman, H. H., Jacova, C. & Massoud, F. Definitions of dementia and predementia states in Alzheimer’s disease and vascular cognitive impairment: consensus from the Canadian conference on diagnosis of dementia. *Alzheimer’s Res. Ther.* **5**, S2 (2013). 24565215 10.1186/alzrt198 PMC3981054

[55] Jak, A. J. et al. Quantification of five neuropsychological approaches to defining mild cognitive impairment. *Am. J. Geriatr. Psychiatry* **17**, 368–375 (2009). 19390294 10.1097/JGP.0b013e31819431d5 PMC2743175

[56] van der Flier, W. M. & Scheltens, P. Epidemiology and risk factors of dementia. *J. Neurol. Neurosurg. Psychiatry* **76**, v2–v7 (2005). 16291918 10.1136/jnnp.2005.082867 PMC1765715

[57] Hays, R. D., Morales, L. S. & Reise, S. P. Item response theory and health outcomes measurement in the 21st century. *Med Care* **38**, II28–II42 (2000). 10982088 10.1097/00005650-200009002-00007 PMC1815384

[58] Liu, M., Harbaugh, A. G., Harring, J. R. & Hancock, G. R. The effect of extreme response and non-extreme response styles on testing measurement invariance. *Front Psychol.* **8**, 726 (2017). 28588521 10.3389/fpsyg.2017.00726 PMC5440768

[59] Schuster, C. & Lubbe, D. A note on residual M-distances for identifying aberrant response patterns. *Br. J. Math. Stat. Psychol.* **73**, 164–169 (2020). 30756381 10.1111/bmsp.12161

[60] The number of Guttman errors as a simple and powerful person-fit statistic - Rob R. Meijer 10.1177/01466216940180 0402 (1994).

[61] Meijer, R. R. & Sijtsma, K. Methodology review: evaluating person fit. *Appl. Psychol. Meas.* **25**, 107–135 (2001).

[62] TabPFN: one model to rule them all? https://arxiv.org/html/2505.20003v1.

[63] Purucker, L. finetune_tabpfn_v2: Code for finetuning TabPFN on one downstream tabular dataset. https://github.com/LennartPurucker/finetune_tabpfn_v2 (2025).

[64] Bengio, Y., Louradour, J., Collobert, R. & Weston, J. Curriculum learning. in *Proc. 26th Annual International Conference on Machine Learning* 41–48 10.1145/1553374.1553380.(Association for Computing Machinery, 2009).

[65] Micikevicius, P. et al. Mixed Precision Training. In *Proc. International Conference on Learning Representations* (2018).

[66] Pascanu, R., Mikolov, T. & Bengio, Y. On the difficulty of training recurrent neural networks. In *Proc. 30th International Conference on Machine Learning* 1310–1318 (PMLR, 2013).

[67] Ott, M., Edunov, S., Grangier, D. & Auli, M. Scaling neural machine translation. In *Proceedings of the Third Conference on Machine Translation: Research Papers* (eds. Bojar, O. et al.) 1–9 (Association for Computational Linguistics, Brussels, Belgium, 2018). 10.18653/v1/W18-6301.

[68] Loshchilov, I. & Hutter, F. Decoupled weight decay regularization. In *Proc. International Conference on Learning Representations* (ICLR, 2018).

[69] Bates, D., Mächler, M., Bolker, B. & Walker, S. Fitting linear mixed-effects models using lme4. *J. Stat. Softw.* **67**, 1–48 (2015).

[70] Lüdecke, D., Ben Shachar, M., Patil, I., Waggoner, P. & Makowski, D. Performance: an R package for assessment, comparison and testing of statistical models. * J. Open Source Softw.* **6**, 3139 (2021).

[71] Riley, R. D. et al. Importance of sample size on the quality and utility of AI-based prediction models for healthcare. *Lancet Digit. Health* **7**, 100857 (2025). 10.1016/j.landig.2025.01.013 40461350

[72] Chen, T. & Guestrin, C. XGBoost: a scalable tree boosting system. in *Proc. 22nd ACM SIGKDD International Conference on Knowledge Discovery and Data Mining* 785–794 10.1145/2939672.2939785.(Association for Computing Machinery, 2016).

[73] Ke, G. et al. LightGBM: A Highly Efficient Gradient Boosting Decision Tree. in *Advances in Neural Information Processing Systems* vol. 30 (Curran Associates, Inc., 2017).

[74] Prokhorenkova, L., Gusev, G., Vorobev, A., Dorogush, A. V. & Gulin, A. CatBoost: unbiased boosting with categorical features. in *Advances in Neural Information Processing Systems* vol. 31 (Curran Associates, Inc., 2018).

[75] Conneau, A. et al. Unsupervised Cross-lingual Representation Learning at Scale. in *Proceedings of the 58th Annual Meeting of the Association for Computational Linguistics* (eds. Jurafsky, D., Chai, J., Schluter, N. & Tetreault, J) 8440–8451 10.18653/v1/2020.acl-main.747.(Association for Computational Linguistics, 2020).

[76] Speer, R. rspeer/wordfreq: v3.0. Zenodo 10.5281/zenodo.7199437(2022).

[77] Lundberg, S. M. & Lee, S.-I. A unified approach to interpreting model predictions. in *Proc. 31st International Conference on Neural Information Processing Systems* 4768–4777 (Curran Associates Inc., 2017).

[78] Vickers, A. J. & Elkin, E. B. Decision curve analysis: a novel method for evaluating prediction models. *Med Decis. Mak.* **26**, 565–574 (2006). 10.1177/0272989X06295361 PMC2577036 17099194

[79] Platt, J. Probabilistic outputs for support vector machines and comparisons to regularized likelihood methods. in *Advances in large margin classifiers* 61–74 (MIT Press, 1999).

[80] Gao, H. Low-Burden AI Approach for Cross-National Early Identification of Cognitive Impairment Using Real-World Questionnaire Response Behaviours. cross-national-cognitive-ai, Zenodo, 10.5281/zenodo.20651481 (2026).42660937

