# Supplementary material for: Low-burden AI approach for cross-national early identification of cognitive impairment using real-world questionnaire response behaviours
Source: Nat Commun. 2026 Jul 28;17:9157. doi: 10.1038/s41467-026-76071-9 (PMC13522467; doi:10.1038/s41467-026-76071-9)
Supplement: Supplementary file 1 [file 41467_2026_76071_MOESM1_ESM.pdf]

Low-Burden AI Approach for Cross-National Early Identification of Cognitive Impairment Using Real-World Questionnaire Response Behaviours

Table of Contents

Supplementary Table 1 2
Supplementary Table 2 3
Supplementary Table 3 4
Supplementary Table 4 5
Supplementary Table 5 6
Supplementary Table 6 7
Supplementary Table 7 7
Supplementary Table 8 8
Supplementary Table 9 10
Supplementary Table 10 10
Supplementary Fig. 1 11
Supplementary Fig. 2 11
Supplementary Fig. 3 12
Supplementary Fig. 4 13
Supplementary Note 1 13
Supplementary References 14

Supplementary Table 1. Respondent characteristics across five national ageing cohorts.

Characteristics	Overall, n (%)	HRS Wave 13 (2016), n (%)	ELSA Wave 7 (2014-15) and wave 8 (2016-17), n (%)	LASI Wave 1 (2017-19), n (%)	MHAS Wave 4 (2015), n (%)	CHARLS Wave 4 (2018), n (%)
Total Respondents	45,604	3,356	5,886	21,141	7,827	7,394
**Age Groups**						
65-69	16,842 (36.9)	847 (25.2)	1,486 (25.2)	8,856 (41.9)	2,563 (32.7)	3,090 (41.8)
70-74	12,074 (26.5)	662 (19.7)	1,570 (26.7)	5,719 (27.1)	2,200 (28.1)	1,923 (26.0)
75-79	8,037 (17.6)	831 (24.8)	1,128 (19.2)	3,351 (15.9)	1,454 (18.6)	1,273 (17.2)
80-84	5,197 (11.4)	589 (17.6)	1,039 (17.7)	1,887 (8.9)	961 (12.3)	721 (9.8)
85-89	2,342 (5.1)	303 (9.0)	475 (8.1)	830 (3.9)	443 (5.7)	291 (3.9)
90+	1,112 (2.4)	124 (3.7)	188 (3.2)	498 (2.4)	206 (2.6)	96 (1.3)
**Sex**						
Male	21,569 (47.3)	1,333 (39.7)	2,587 (44.0)	10,413 (49.3)	3,645 (46.6)	3,591 (48.6)
Female	24,035 (52.7)	2,023 (60.3)	3,299 (56.0)	10,728 (50.7)	4,182 (53.4)	3,803 (51.4)
**Harmonised Education Level**						
Less than upper secondary	32,994 (72.3)	541 (16.1)	1,768 (30.0)	16,775 (79.3)	7,028 (89.8)	6,882 (93.1)
Upper secondary and vocational	9,213 (20.2)	1,999 (59.6)	3,108 (52.8)	3,527 (16.7)	189 (2.4)	390 (5.3)
Tertiary	3,397 (7.4)	816 (24.3)	1,010 (17.2)	839 (4.0)	610 (7.8)	122 (1.6)
**Cognitive Status**						
Normal	25,291 (55.5)	2,851 (85.0)	5,016 (85.2)	9,248 (43.7)	4,260 (54.4)	3,916 (53.0)
MCI risk	13,888 (30.5)	418 (12.5)	658 (11.2)	8,047 (38.1)	2,372 (30.3)	2,393 (32.4)
Dementia risk	6,425 (14.1)	87 (2.6)	212 (3.6)	3,846 (18.2)	1,195 (15.3)	1,085 (14.7)

HRS = Health and Retirement Study (United States); ELSA = English Longitudinal Study of Ageing (England); LASI = Longitudinal Ageing Study in India (India); MHAS = Mexican Health and Ageing Study (Mexico); CHARLS = China Health and Retirement Longitudinal Study (China); MCI = Mild Cognitive Impairment.

Supplementary Table 2. Psychosocial questionnaires used for generating low-quality response (LQR) indicators across cohorts.

	HRS			ELSA			MHAS			LASI			CHARLS		
**No.**	**Label**	**# items**	**# resp. options**	**Label**	**# items**	**# resp. options**	**Label**	**# items**	**# resp. options**	**Label**	**# items**	**# resp. options**	**Label**	**# items**	**# resp. options**
1	Diener/Satisfaction with life	5	7	Cultural participation	4	6	Locus of control scale - Luck	4	4	Everyday Discrimination	6	6	CES-D subscale - Affect/Mood	5	4
2	Loneliness	3	3	Diener/Satisfaction with life	5	7	Locus of control scale - Responsibility	3	4	Satisfaction with life	5	7	CES-D subscale - Somatic and retarded activity	5	4
3	Neighborhood disorder	4	7	Loneliness	5	3	Diener/Satisfaction with life	5	3						
4	Neighborhood cohesion	4	7	CASP-19 Control	6	4	Personality	6	4						
5	Perceived constraints on personal control	5	6	CASP-19 Autonomy	5	4									
6	Perceived mastery	5	6	CASP-19 Pleasure	4	4									
7	Religiosity	4	6	CASP-19 Self-realisation	4	4									
8	Perceived discrimination	5	6												
9	Big5 Extraversion	5	4												
10	Big5 Agreeableness	5	4												
11	Big5 Neuroticism	4	4												
12	Big5 Conscientiousness	5	4												
13	Big5 Openness	7	4												
14	Purpose in life	7	6												

Supplementary Table 3. Description of LQR indicators.

Indicator	Rationale	Computation
Item non-response	Missing data in surveys are a frequent concern in research on aging [1].When respondents skip individual survey items (e.g., implicit don't know answers [2]) or provide only partial data when completing a longer questionnaire battery, the resulting missing values reduce statistical power and can potentially bias the validity of conclusions from the survey [3].	We calculated the proportion of missing values per respondent and questionnaire in each study using SAS version 9.4 (Cary, NJ). The scores were arcsine transformed to stabilize the variance of the calculated proportions.
Response inconsistency	According to classical test theory, random measurement error reduces the reliability of scores and the amount of true score variance in survey data. While there are many potential sources of measurement error, it can result from participants giving noisy responses because they are uncertain about their answers or because they are engaging in mental coin flipping (i.e., selecting responses at random) as one form of survey satisficing [4].	To derive an index of random measurement error around each person's true questionnaire scores, we first transformed all survey items onto a common 0-10 metric. We then applied a single-factor model to each questionnaire, saved the residual errors (i.e., the difference between a person's observed and predicted scores for each item), and computed the variance of the item residuals for each person. The factor models were estimated using M*plus* version 7.11 [5].Missing values were accommodated with full information maximum likelihood estimation. The residual error variances were square root transformed to normalize their distribution.
Guttman errors	Modern test theory provides so-called person-fit statistics as a means to determining if a person's response pattern is likely inaccurate on the basis of an item response theory (IRT) model. The number of Guttman errors represents the simplest nonparametric person-fit statistic [6]. Unlike parametric person-fit measures, examining Guttman errors does not require that a parametric IRT model fits the data [7]. Items are ordered according to their difficulty levels, and Guttman errors quantify the extent to which a person's response pattern does not conform to the expected pattern given the item difficulty levels.	We used the R package PerFit [8] to obtain the number of Guttman errors for ordered categorical (i.e., polytomous) responses per questionnaire in each study. The program accommodates missing values via Hotdeck imputation. The Guttman errors were normed to a 0-1 range using the recursive algorithm by Emons (2008) [9] and log-transformed (a constant of 0.1 was added to allow log-transformation of zero values).
Aberrant response pattern	Outliers are responses that deviate to a large extent from those of other respondents. Invalid outlier responses can have a large biasing impact on the results of empirical analyses. The squared Mahalanobis distance (M-distance) represents an extension of univariate (single-variable) outlier statistics to multivariate responses [10]. It is frequently used to screen for anomalous response patterns and has been shown to represent a sensitive indicator of survey satisficing [11,12].	We computed the squared Mahalanobis distance of each respondent's response pattern per questionnaire using M*plus.* Missing values were accommodated with maximum likelihood estimation. To place the Mahalanobis distances on a common (0 to 1) metric for each questionnaire, they were transformed using the chi-square cumulative distribution function.
Extreme response	Extreme responding refers to a tendency to select the highest and lowest (i.e., extreme) response options regardless of question content. Extreme response bias can attenuate or inflate relationships in empirical research [13].	Extreme responding was calculated per questionnaire using a two-dimensional nominal response model (NRM) [14]. The NRM addresses limitations of methods that merely count the number of times that a participant selects an extreme response. Whereas these methods confound the measurement of response behaviours and a participant's extremeness on the latent trait measured, the NRM separates these two aspects by extracting them as two separate latent factors from the response pattern. The NRM was estimated in M*plus* using maximum likelihood estimation [15].
lz person-fit statistic	Parametric person-fit statistics provide a model-based assessment of whether an individual's response pattern is consistent with expectations under an item response theory (IRT) model. The *lz* statistic quantifies the extent to which a respondent's pattern deviates from the most likely pattern given their estimated trait level, capturing global misfit.	Computed using the R package *PerFit* for polytomous responses per questionnaire. Missing values were handled via hot-deck imputation. Scores were normalized to a 0–1 range and log-transformed using the same procedure applied to Guttman errors.
U3 person-fit statistic	The U3 statistic provides a nonparametric measure of aberrant responding based on the number and structure of Guttman errors in a response pattern. It captures the extent to which a respondent's answers violate the expected ordering of items, offering a complementary perspective to model-based fit indices.	Computed using the R package *PerFit* for polytomous responses per questionnaire. Missing values were handled via hot-deck imputation. Scores were normalized to a 0–1 range and log-transformed using the same procedure applied to Guttman errors.

Supplementary Table 4. Survey languages used in five national ageing cohorts.

Cohort (Country)	No.	Language Category
ELSA(England)	1	English
MHAS (Mexico)	2	Spanish
CHARLS (China)	3	Chinese
HRS (US)	4	English
	5	Spanish
LASI (India)	6	English
	7	Hindi
	8	Kannada
	9	Konkani
	10	Malayalam
	11	Gujarati
	12	Tamil
	13	Punjabi
	14	Manipuri
	15	Mizo
	16	Urdu
	17	Garo
	18	Khasi
	19	Bengali
	20	Assamese
	21	Odiya
	22	Marathi
	23	Telugu

Supplementary Table 5. AUROC for identifying current cognitive impairment risk by questionnaire and country: cross-national model with default vs. fine-tuned weights.

Cohort (Country)	Questionnaire	LQR-only Default [95% CI]	LQR-only Fine-tuned [95% CI]	Model I Default [95% CI]	Model I Fine-tuned [95% CI]	Model II Default [95% CI]	Model II Fine-tuned [95% CI]
HRS (US)	1	0.734 [0.694, 0.771]	0.735 [0.695, 0.774]	0.786 [0.744, 0.825]	0.804 [0.766, 0.84]	0.856 [0.826, 0.884]	0.866 [0.838, 0.893]
	2	0.655 [0.611, 0.697]	0.651 [0.606, 0.692]	0.781 [0.738, 0.818]	0.786 [0.744, 0.822]	0.849 [0.818, 0.878]	0.857 [0.827, 0.886]
	3	0.71 [0.665, 0.758]	0.706 [0.661, 0.755]	0.796 [0.753, 0.833]	0.805 [0.766, 0.842]	0.852 [0.821, 0.88]	0.863 [0.832, 0.893]
	4	0.706 [0.661, 0.751]	0.699 [0.656, 0.743]	0.79 [0.747, 0.826]	0.801 [0.763, 0.837]	0.85 [0.818, 0.879]	0.855 [0.824, 0.886]
	5	0.705 [0.662, 0.749]	0.709 [0.666, 0.752]	0.787 [0.744, 0.825]	0.796 [0.755, 0.833]	0.857 [0.828, 0.884]	0.865 [0.837, 0.892]
	6	0.658 [0.613, 0.704]	0.662 [0.618, 0.709]	0.784 [0.743, 0.822]	0.794 [0.758, 0.831]	0.854 [0.823, 0.882]	0.859 [0.83, 0.888]
	7	0.543 [0.489, 0.594]	0.577 [0.525, 0.627]	0.767 [0.723, 0.806]	0.765 [0.724, 0.806]	0.846 [0.815, 0.877]	0.852 [0.82, 0.883]
	8	0.632 [0.584, 0.679]	0.617 [0.567, 0.666]	0.781 [0.738, 0.818]	0.782 [0.74, 0.819]	0.853 [0.824, 0.881]	0.862 [0.833, 0.89]
	9	0.692 [0.649, 0.737]	0.688 [0.645, 0.735]	0.786 [0.742, 0.822]	0.798 [0.757, 0.833]	0.853 [0.822, 0.882]	0.863 [0.832, 0.892]
	10	0.724 [0.681, 0.766]	0.726 [0.684, 0.769]	0.795 [0.754, 0.831]	0.809 [0.771, 0.843]	0.857 [0.828, 0.886]	0.866 [0.836, 0.894]
	11	0.691 [0.645, 0.735]	0.687 [0.64, 0.732]	0.781 [0.738, 0.818]	0.79 [0.75, 0.825]	0.851 [0.82, 0.879]	0.858 [0.828, 0.887]
	12	0.782 [0.743, 0.821]	0.778 [0.739, 0.819]	0.808 [0.767, 0.842]	0.829 [0.793, 0.863]	0.86 [0.83, 0.888]	0.872 [0.841, 0.899]
	13	0.737 [0.7, 0.777]	0.739 [0.701, 0.778]	0.789 [0.746, 0.826]	0.804 [0.767, 0.84]	0.857 [0.827, 0.884]	0.867 [0.839, 0.895]
	14	0.757 [0.715, 0.796]	0.754 [0.711, 0.792]	0.803 [0.762, 0.837]	0.822 [0.785, 0.853]	0.862 [0.832, 0.889]	0.873 [0.844, 0.901]
ELSA (England)	1	0.613 [0.576, 0.647]	0.628 [0.594, 0.661]	0.735 [0.706, 0.763]	0.735 [0.708, 0.764]	0.816 [0.792, 0.84]	0.82 [0.796, 0.845]
	2	0.659 [0.624, 0.691]	0.662 [0.626, 0.694]	0.743 [0.716, 0.771]	0.751 [0.724, 0.779]	0.826 [0.804, 0.851]	0.833 [0.812, 0.857]
	3	0.715 [0.687, 0.745]	0.717 [0.689, 0.746]	0.755 [0.726, 0.781]	0.773 [0.747, 0.799]	0.828 [0.805, 0.852]	0.839 [0.818, 0.862]
	4	0.706 [0.674, 0.738]	0.706 [0.674, 0.738]	0.747 [0.719, 0.775]	0.762 [0.735, 0.791]	0.827 [0.803, 0.851]	0.835 [0.812, 0.858]
	5	0.693 [0.657, 0.728]	0.688 [0.653, 0.724]	0.748 [0.72, 0.776]	0.761 [0.734, 0.79]	0.824 [0.8, 0.848]	0.833 [0.809, 0.857]
	6	0.679 [0.648, 0.712]	0.689 [0.657, 0.722]	0.743 [0.715, 0.769]	0.761 [0.734, 0.787]	0.823 [0.799, 0.848]	0.832 [0.809, 0.856]
	7	0.685 [0.647, 0.721]	0.701 [0.665, 0.734]	0.748 [0.719, 0.775]	0.763 [0.734, 0.79]	0.825 [0.801, 0.849]	0.834 [0.812, 0.857]
LASI (India)	1	0.612 [0.599, 0.624]	0.624 [0.612, 0.636]	0.829 [0.819, 0.838]	0.836 [0.826, 0.845]	0.836 [0.826, 0.845]	0.843 [0.834, 0.852]
	2	0.623 [0.61, 0.635]	0.622 [0.609, 0.635]	0.823 [0.813, 0.832]	0.826 [0.816, 0.835]	0.84 [0.831, 0.849]	0.847 [0.838, 0.855]
MHAS (Mexico)	1	0.706 [0.686, 0.725]	0.705 [0.685, 0.724]	0.71 [0.691, 0.729]	0.743 [0.725, 0.761]	0.714 [0.694, 0.732]	0.731 [0.712, 0.749]
	2	0.564 [0.547, 0.581]	0.563 [0.546, 0.58]	0.679 [0.66, 0.698]	0.687 [0.667, 0.706]	0.683 [0.663, 0.702]	0.68 [0.66, 0.699]
	3	0.566 [0.547, 0.587]	0.56 [0.541, 0.58]	0.675 [0.655, 0.695]	0.682 [0.663, 0.702]	0.682 [0.662, 0.702]	0.683 [0.663, 0.703]
	4	0.614 [0.593, 0.635]	0.613 [0.591, 0.634]	0.685 [0.665, 0.703]	0.695 [0.674, 0.714]	0.69 [0.669, 0.708]	0.692 [0.672, 0.711]
CHARLS (China)	1	0.662 [0.64, 0.684]	0.658 [0.636, 0.68]	0.711 [0.692, 0.731]	0.714 [0.694, 0.734]	0.714 [0.695, 0.733]	0.717 [0.698, 0.737]
	2	0.653 [0.632, 0.674]	0.652 [0.631, 0.673]	0.707 [0.688, 0.727]	0.714 [0.695, 0.733]	0.715 [0.697, 0.735]	0.718 [0.699, 0.738]

AUROC = Area Under the Receiver Operating Characteristic curve; CI = Confidence Interval.

Supplementary Table 6. Detailed descriptions of cognitively demanding daily activity variables.

Cohort	Variable	Original question	Response Options
HRS	Email use	Do you regularly use the Internet (or the World Wide Web) for sending and receiving e-mail or for any other purpose, such as making purchases, searching for information, or making travel reservations?	Yes / No
	Driving status	Are you able to drive?	Yes / No
ELSA	Internet/email use	I use the internet and/or email	Yes / No
	Frequency of internet/email use	On average, how often do you use the internet or email	Every day, at least weekly, at least monthly, every 3 months, less than every 3 months, never

Supplementary Table 7. Prospective versus concurrent predictive performance of cross-national models for cognitive impairment.

Dataset	Timepoint	Model I (AUROC)	Model II (AUROC)
Four-country	Earlier-wave where available	0.74	0.82
Four-country	Current	0.77	0.82
CHARLS	Earlier-wave	0.68	0.69
CHARLS	Current	0.71	0.72

AUROC = area under the receiver operating characteristic curve. Model I includes LQR indicators and demographics; Model II additionally includes cognitively demanding activity features (available in HRS and ELSA only). The four-country dataset comprises HRS, ELSA, LASI, and MHAS. In prospective validation analyses, earlier-wave predictors were used where available, whereas LASI contributed concurrent measures from wave 1 only. CHARLS served as external validation.

Supplementary Table 8. TRIPOD+AI checklist for reporting artificial intelligence–based prediction model development and validation.

Section/Topic	Item	Development / evaluation[1]	Checklist item	Reported on page
**TITLE**				
Title	1	D;E	Identify the study as developing or evaluating the performance of a multivariable prediction model, the target population, and the outcome to be predicted	1
**ABSTRACT**				
Abstract	2	D;E	See TRIPOD+AI for Abstracts checklist	2
**INTRODUCTION**				
Background	3a	D;E	Explain the healthcare context (including whether diagnostic or prognostic) and rationale for developing or evaluating the prediction model, including references to existing models	2-4
	3b	D;E	Describe the target population and the intended purpose of the prediction model in the context of the care pathway, including its intended users (e.g., healthcare professionals, patients, public)	2-4, 15-16
	3c	D;E	Describe any known health inequalities between sociodemographic groups	2-3
Objectives	4	D;E	Specify the study objectives, including whether the study describes the development or validation of a prediction model (or both)	4
**METHODS**				
Data	5a	D;E	Describe the sources of data separately for the development and evaluation datasets (e.g., randomised trial, cohort, routine care or registry data), the rationale for using these data, and representativeness of the data	18, Supplementary Table 1
	5b	D;E	Specify the dates of the collected participant data, including start and end of participant accrual; and, if applicable, end of follow-up	18, Supplementary Table 1
Participants	6a	D;E	Specify key elements of the study setting (e.g., primary care, secondary care, general population) including the number and location of centres	18, Supplementary Table 1
	6b	D;E	Describe the eligibility criteria for study participants	18
	6c	D;E	Give details of any treatments received, and how they were handled during model development or evaluation, if relevant	N/A
Data preparation	7	D;E	Describe any data pre-processing and quality checking, including whether this was similar across relevant sociodemographic groups	20-25, Supplementary Table 10
Outcome	8a	D;E	Clearly define the outcome that is being predicted and the time horizon, including how and when assessed, the rationale for choosing this outcome, and whether the method of outcome assessment is consistent across sociodemographic groups	18-20, Supplementary Table 9
	8b	D;E	If outcome assessment requires subjective interpretation, describe the qualifications and demographic characteristics of the outcome assessors	N/A
	8c	D;E	Report any actions to blind assessment of the outcome to be predicted	N/A
Predictors	9a	D	Describe the choice of initial predictors (e.g., literature, previous models, all available predictors) and any pre-selection of predictors before model building	21
	9b	D;E	Clearly define all predictors, including how and when they were measured (and any actions to blind assessment of predictors for the outcome and other predictors)	21, Supplementary Tables 2-4, 6
	9c	D;E	If predictor measurement requires subjective interpretation, describe the qualifications and demographic characteristics of the predictor assessors	N/A
Sample size	10	D;E	Explain how the study size was arrived at (separately for development and evaluation), and justify that the study size was sufficient to answer the research question. Include details of any sample size calculation	18, 23, Supplementary Tables 1, 10
Missing data	11	D;E	Describe how missing data were handled. Provide reasons for omitting any data	22-23, Supplementary Table 10
Analytical methods	12a	D	Describe how the data were used (e.g., for development and evaluation of model performance) in the analysis, including whether the data were partitioned, considering any sample size requirements	23-24
	12b	D	Depending on the type of model, describe how predictors were handled in the analyses (functional form, rescaling, transformation, or any standardisation).	20-21, 24-25
	12c	D	Specify the type of model, rationale[2], all model-building steps, including any hyperparameter tuning, and method for internal validation	21-23
	12d	D;E	Describe if and how any heterogeneity in estimates of model parameter values and model performance was handled and quantified across clusters (e.g., hospitals, countries). See TRIPOD-Cluster for additional considerations[3]	22-23, 25
	12e	D;E	Specify all measures and plots used (and their rationale) to evaluate model performance (e.g., discrimination, calibration, clinical utility) and, if relevant, to compare multiple models	23, 25-26
	12f	E	Describe any model updating (e.g., recalibration) arising from the model evaluation, either overall or for particular sociodemographic groups or settings	9, 21-22, 24
	12g	E	For model evaluation, describe how the model predictions were calculated (e.g., formula, code, object, application programming interface)	21-23, 25-26
Class imbalance	13	D;E	If class imbalance methods were used, state why and how this was done, and any subsequent methods to recalibrate the model or the model predictions	22-23
Fairness	14	D;E	Describe any approaches that were used to address model fairness and their rationale	10-12, 15, 25, Supplementary Note 1
Model output	15	D	Specify the output of the prediction model (e.g., probabilities, classification). Provide details and rationale for any classification and how the thresholds were identified	6, 18-20, 23, 25-26

Training versus evaluation	16	D;E	Identify any differences between the development and evaluation data in healthcare setting, eligibility criteria, outcome, and predictors	7, 22-23
Ethical approval	17	D;E	Name the institutional research board or ethics committee that approved the study and describe the participant-informed consent or the ethics committee waiver of informed consent	18
**OPEN SCIENCE**				
Funding	18a	D;E	Give the source of funding and the role of the funders for the present study	28
Conflicts of interest	18b	D;E	Declare any conflicts of interest and financial disclosures for all authors	28
Protocol	18c	D;E	Indicate where the study protocol can be accessed or state that a protocol was not prepared	N/A
Registration	18d	D;E	Provide registration information for the study, including register name and registration number, or state that the study was not registered	N/A
Data sharing	18e	D;E	Provide details of the availability of the study data	27
Code sharing	18f	D;E	Provide details of the availability of the analytical code[4]	27
**PATIENT & PUBLIC INVOLVEMENT**				
Patient & Public Involvement	19	D;E	Provide details of any patient and public involvement during the design, conduct, reporting, interpretation, or dissemination of the study or state no involvement.	N/A
**RESULTS**				
Participants	20a	D;E	Describe the flow of participants through the study, including the number of participants with and without the outcome and, if applicable, a summary of the follow-up time. A diagram may be helpful.	6-7, 18, 23, Supplementary Tables 1, 10
	20b	D;E	Report the characteristics overall and, where applicable, for each data source or setting, including the key dates, key predictors (including demographics), treatments received, sample size, number of outcome events, follow-up time, and amount of missing data. A table may be helpful. Report any differences across key demographic groups.	6-7, Supplementary Table 1
	20c	E	For model evaluation, show a comparison with the development data of the distribution of important predictors (demographics, predictors, and outcome).	Supplementary Tables 1, 10
Model development	21	D;E	Specify the number of participants and outcome events in each analysis (e.g., for model development, hyperparameter tuning, model evaluation)	6-7, 23, Supplementary Table 10
Model specification	22	D	Provide details of the full prediction model (e.g., formula, code, object, application programming interface) to allow predictions in new individuals and to enable third-party evaluation and implementation, including any restrictions to access or re-use (e.g., freely available, proprietary)[5]	21-23, 27
Model performance	23a	D;E	Report model performance estimates with confidence intervals, including for any key subgroups (e.g., sociodemographic). Consider plots to aid presentation.	7-12, Supplementary Tables 5, 7; Supplementary Figs. 1-4
	23b	D;E	If examined, report results of any heterogeneity in model performance across clusters. See TRIPOD Cluster for additional details[3].	8-12, 15
Model updating	24	E	Report the results from any model updating, including the updated model and subsequent performance	9, Supplementary Table 5, Supplementary Note 1
**DISCUSSION**				
Interpretation	25	D;E	Give an overall interpretation of the main results, including issues of fairness in the context of the objectives and previous studies	13-17
Limitations	26	D;E	Discuss any limitations of the study (such as a non-representative sample, sample size, overfitting, missing data) and their effects on any biases, statistical uncertainty, and generalizability	17
Usability of the model in the context of current care	27a	D	Describe how poor quality or unavailable input data (e.g., predictor values) should be assessed and handled when implementing the prediction model	15-16, 22-23
	27b	D	Specify whether users will be required to interact in the handling of the input data or use of the model, and what level of expertise is required of users	15-17
	27c	D;E	Discuss any next steps for future research, with a specific view to applicability and generalizability of the model	15-17

[1] D=items relevant only to the development of a prediction model; E=items relating solely to the evaluation of a prediction model; D;E=items applicable to both the development and evaluation of a prediction model.
[2] Separately for all model building approaches.
[3] TRIPOD-Cluster is a checklist of reporting recommendations for studies developing or validating models that explicitly account for clustering or explore heterogeneity in model performance (eg, at different hospitals or centres). Debray et al, BMJ 2023; 380: e071018 [DOI: 10.1136/bmj-2022-071018]
[4] This relates to the analysis code, for example, any data cleaning, feature engineering, model building, evaluation.
[5] This relates to the code to implement the model to get estimates of risk for a new individual.

Supplementary Table 9. Mixed-effects logistic regression models examining associations of age and educational attainment with cognitive impairment.

This supplementary table presents results from mixed-effects logistic regression models examining associations between age, educational attainment, and cognitive impairment under different classification approaches. Age was standardised, and models included country-level random intercepts to account for cross-national clustering. When cognitive impairment status was derived using the multilevel latent class approach, increasing age was strongly and positively associated with both overall cognitive impairment (MCI or dementia) and dementia specifically (odds ratios [ORs] ≈1.25–1.26 per standard deviation increase in age). In contrast, when impairment was defined using conventional SD-based thresholds (<1.0 SD or <1.5 SD below the mean), age exhibited an inverse association with cognitive impairment (ORs <1), implying lower odds of impairment at older ages—an epidemiologically implausible pattern.

**Outcome Variable**	**Predictor – Age (Standardised)**[a]	**Predictor – Education Attainment**[a]		
		**Less than upper secondary**	**Upper secondary & vocational**	**Tertiary**
Cognitive Impairment (Dementia+MCI), Latent Class Approach	1.26 (1.23, 1.28)	Reference Level	0.10 (0.09, 0.11)	0.06 (0.05, 0.07)
Dementia, Latent Class Approach	1.25 (1.22, 1.28)		0.15 (0.13, 0.17)	0.09 (0.07, 0.11)
Threshold Classification, <1.0SD	0.94 (0.92, 0.96)		0.15 (0.14, 0.16)	0.07 (0.06, 0.08)
Threshold Classification, < 1.5SD	0.92 (0.90, 0.95)		0.19 (0.17, 0.21)	0.09 (0.08, 0.11)

[a] Odds ratio (OR) and 95% confidence interval (CI)

Supplementary Table 10. Final stacked dataset used for modelling.

The final stacked dataset used for modelling comprised 176,564 observations, with an overall missingness rate across the seven LQR indicators of 2.1%. In addition, 5.8% of questionnaire-specific observations showed item non-response (i.e., asinmiss indicator > 0).

**Cohort (country)**	**Total observations (questionnaire-level)**	**Training set (10% ICL[a])**	**Fine-tuning set (30%)**	**Validation set (25%)**	**Test set (35%)**
HRS (US)	46,984	4,704	14,084	11,746	16,450
ELSA (England)	41,202	4,123	12,348	10,304	14,427
LASI (India)	42,282	4,230	12,682	10,570	14,800
MHAS (Mexico)	31,308	3,132	9,388	7,828	10,960
CHARLS (China; external)	14,788	1,478	N/A	N/A	5,176
Total	176,564	17,667	48,502	40,448	61,813

[a]ICL = In-context learning

Supplementary Fig. 1. Sensitivity analyses of the proposed cross-national model across three predictor sets (LQR predictors alone, Model I, and Model II).

Under the joint-training strategy, the cross-national model's average performance remained robust across 100 random data-splitting repetitions, consistently within ±1 SD of random splits (AUROC Z-scores for cognitive impairment risk: four-country = 0.14; external validation CHARLS = 0.41).

When comparing predictive performance for cognitive impairment with three traditional ensemble models under progressively smaller subsets of the training data (i.e., limited data availability; Fig. 3b), the model consistently demonstrated greater robustness. Specifically, its mean AUROC remained stable on the external CHARLS cohort, consistently close to 0.7 when reducing training proportions from 100% down to 60%; even at 25%, the four-country mean AUROC remained above 0.7. Moreover, the cross-national feature-transfer benefits previously described were also not observed in the ensemble models under joint training.

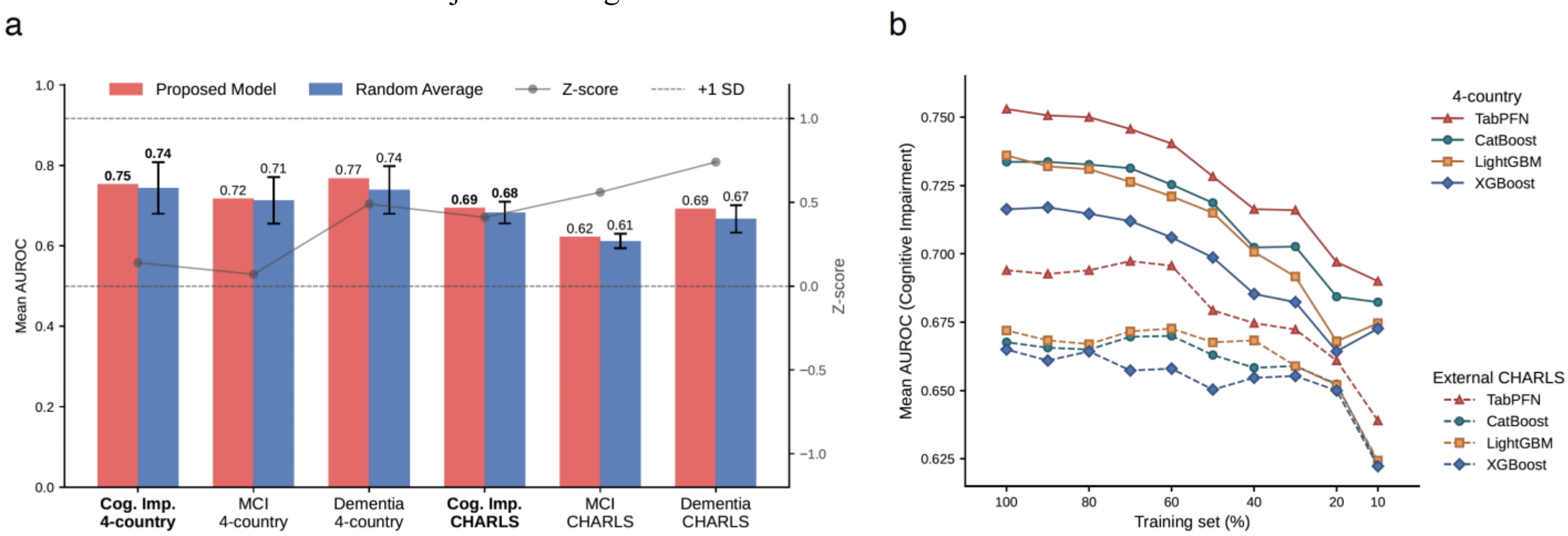

(a) Robustness of the proposed model compared with 100 random data-splitting repetitions; error bars indicate ±1 s.d. across random splits. (b) AUROC performance of the proposed model and three ensemble learning models (CatBoost, LightGBM, XGBoost) across different training set proportions. Independent test sets (35%): four-country, *N* = 13,376 respondents (56,637 questionnaire-specific observations); external CHARLS, *N* = 2,588 respondents (5,176 questionnaire-specific observations). AUROC = area under the receiver operating characteristic curve. Source data are provided as a Source Data file.

Supplementary Fig. 2. External validation on CHARLS: AUROC comparison of cross-national models vs. CHARLS-trained models across questionnaires.

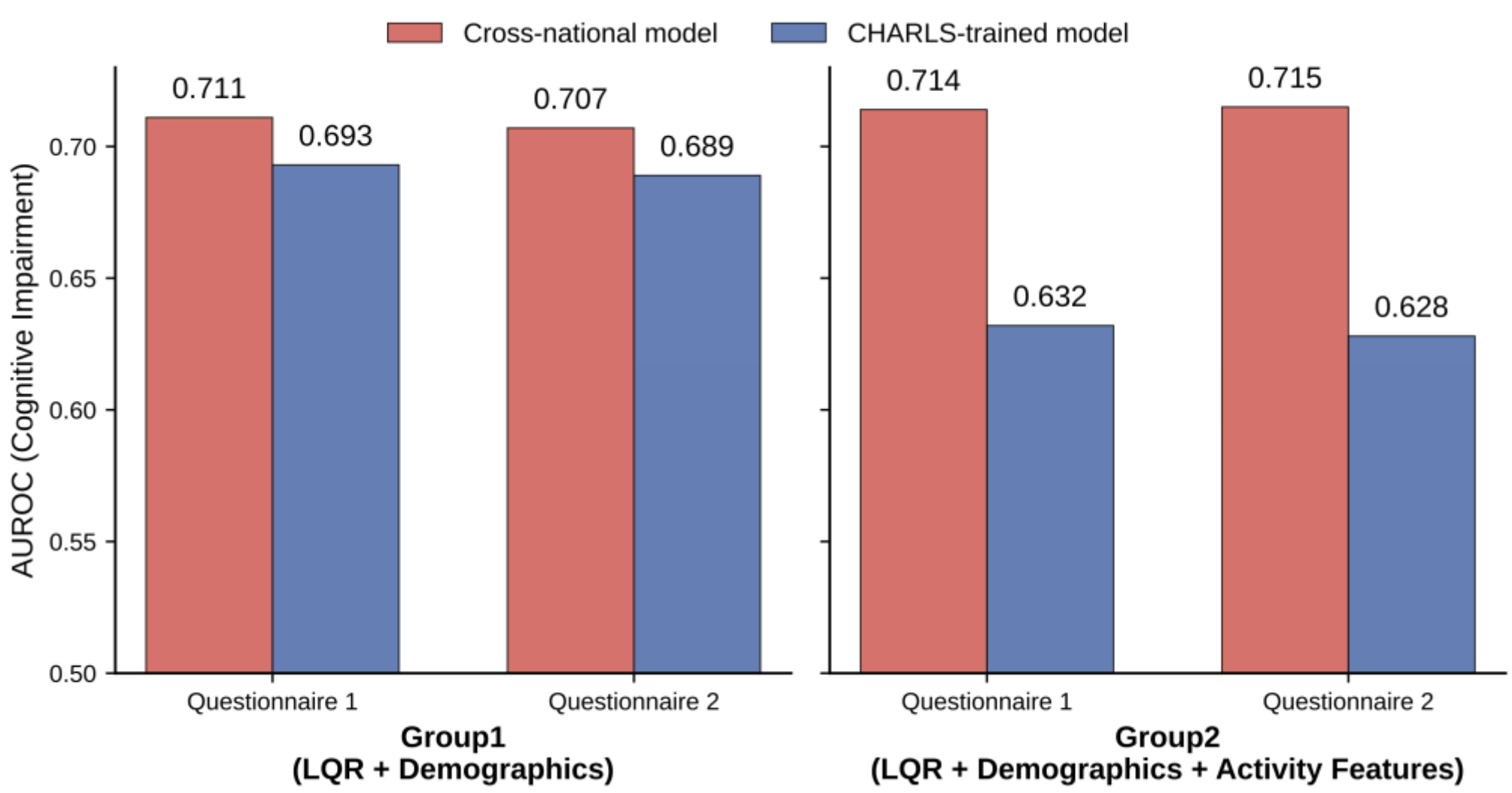

Bar charts show AUROC for cognitive impairment risk identification across two CHARLS questionnaires, comparing the cross-national model with a CHARLS-trained country-specific model, for Group 1 (LQR indicators and demographics) and Group 2 (LQR indicators, demographics, and cross-nationally transferred activity features). External CHARLS test set: N = 2,588 respondents (5,176 questionnaire-specific observations). AUROC = area under the receiver operating characteristic curve; LQR = low-quality response. Source data are provided as a Source Data file.

Supplementary Fig. 3. Cognitive impairment AUROC (LQR-only predictors) by response quality and estimated text complexity of 29 psychosocial questionnaires across countries.

The following analyses were based on models using LQR indicators and survey language only. To further examine questionnaire-level variation in predictive performance, we analysed the relationships between predictive performance, overall response quality, and text complexity across questionnaires. The results showed that questionnaires with stronger predictive performance generally had lower response quality (lower percentile ranks) and moderate-to-high complexity; this trend was particularly evident in HRS, which employed the largest number of questionnaires and had higher overall predictive accuracy as measured by AUROC values. Despite cross-country variations, the best-performing questionnaire within each country typically exhibited a low-quality, high-complexity pattern. Fine-tuning benefits were also generally greater for questionnaires characterised by higher complexity and lower response quality, a pattern that was most evident in HRS (Supplementary Table 5).

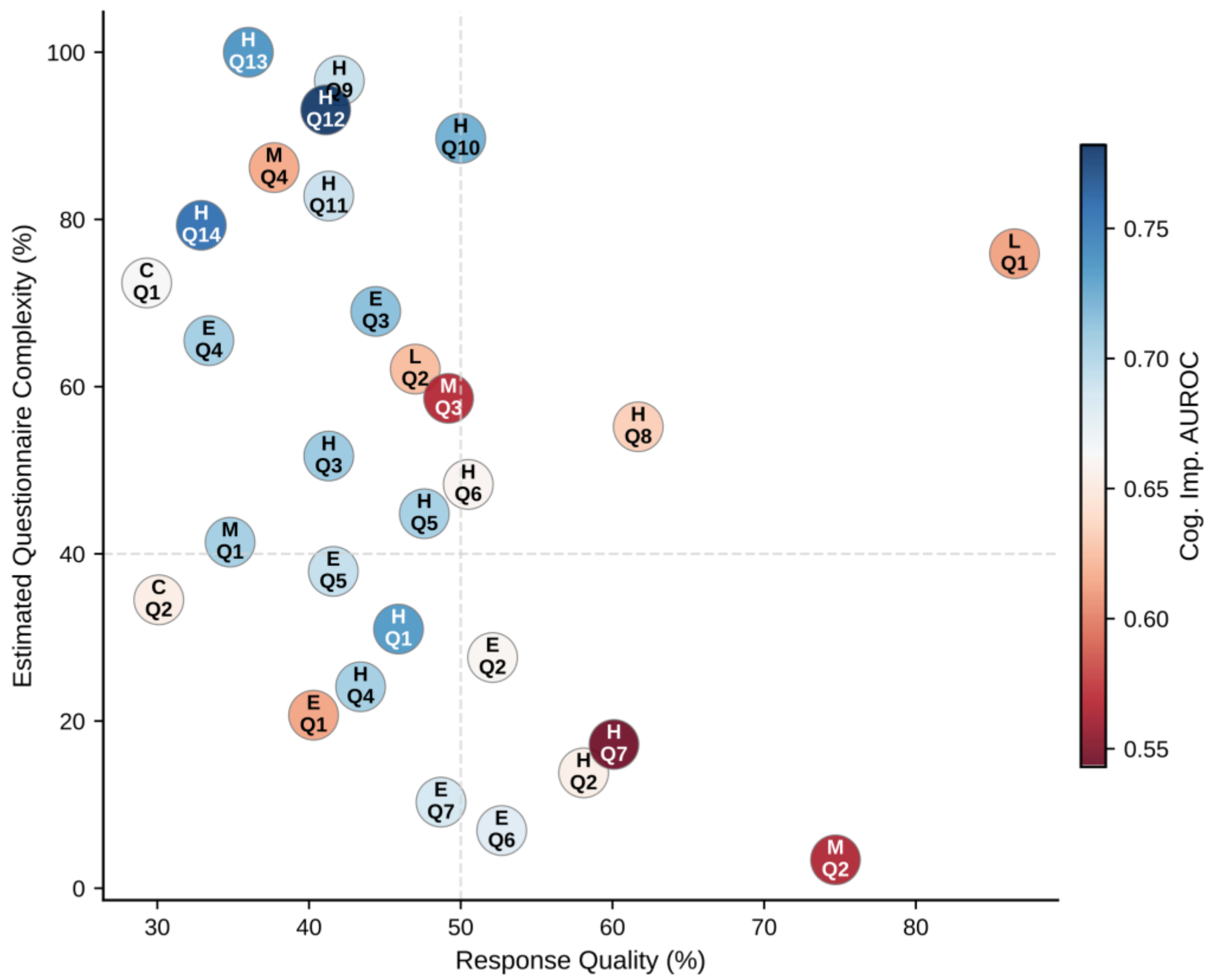

Each bubble represents one of 29 psychosocial questionnaires within each national ageing cohort: HRS (H; US, n = 14), ELSA (E; England, n = 7), LASI (L; India, n = 2), MHAS (M; Mexico, n = 4), CHARLS (C; China, n = 2). Response quality and text complexity are represented as percentile ranks based on the overall distribution across all questionnaires and countries. Bubble colour indicates cognitive impairment AUROC. Text complexity was estimated by aggregating item-level semantic comprehension difficulty, semantic rarity, and difficult word ratio. AUROC = area under the receiver operating characteristic curve; CHARLS = China Health and Retirement Longitudinal Study; ELSA = English Longitudinal Study of Ageing; HRS = Health and Retirement Study; LASI = Longitudinal Ageing Study in India; MHAS = Mexican Health and Ageing Study. Source data are provided as a Source Data file.

Supplementary Fig. 4. Calibration curves for predicted cognitive impairment risk across countries.

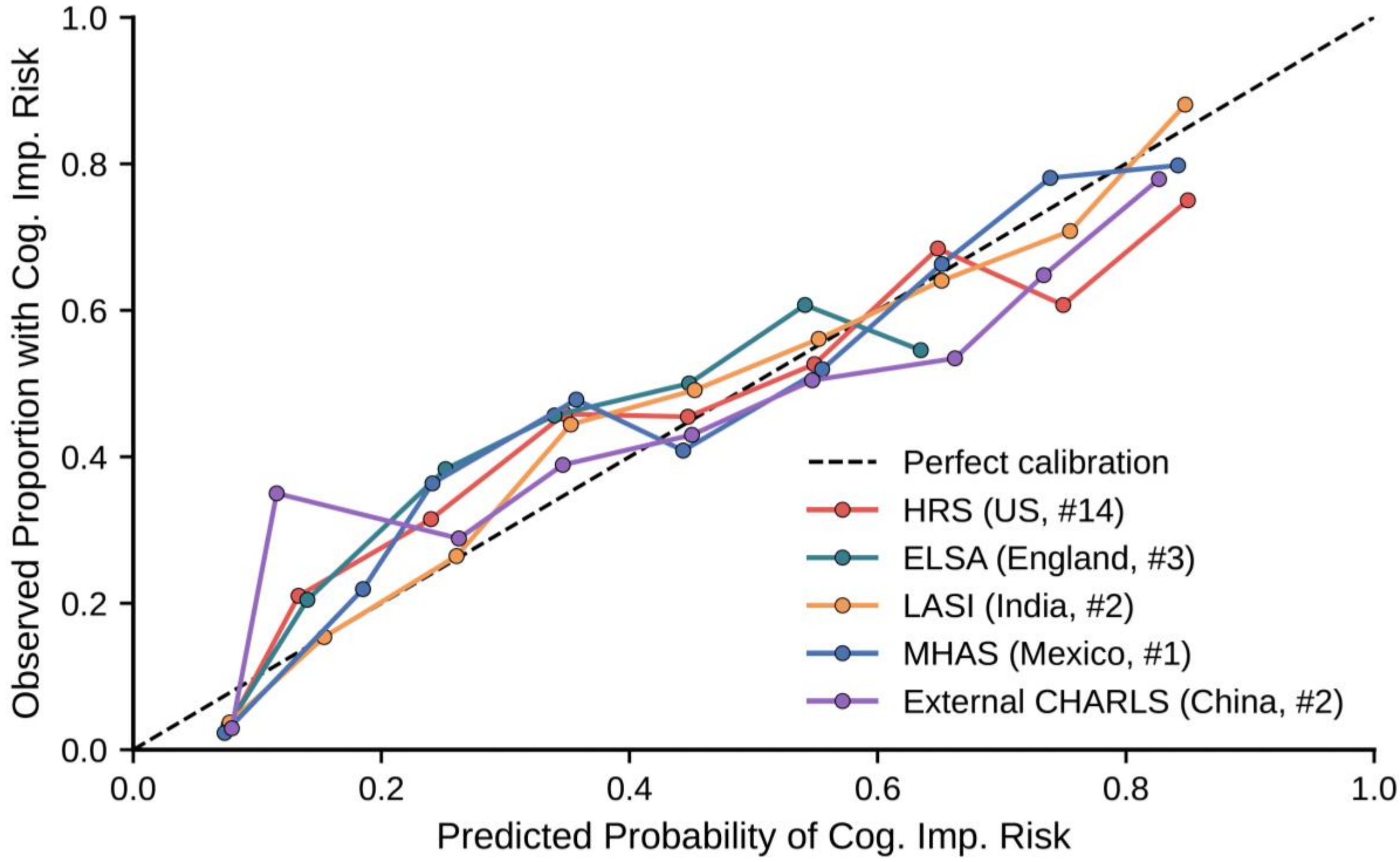

Calibration curves of the cross-national model (using fine-tuned TabPFN weights) after Platt calibration, plotted using the best-performing questionnaire in each cohort (#, questionnaire number). Curves were calculated using 10 probability bins. Independent test sets (35%): HRS, $N = 1,175$; ELSA, $N = 2,061$; LASI, $N = 7,400$; MHAS, $N = 2,740$; external CHARLS, $N = 2,588$. Cog. Imp. = cognitive impairment; CHARLS = China Health and Retirement Longitudinal Study; ELSA = English Longitudinal Study of Ageing; HRS = Health and Retirement Study; LASI = Longitudinal Ageing Study in India; MHAS = Mexican Health and Ageing Study; TabPFN = Tabular Prior-data Fitted Network. Source data are provided as a Source Data file.

Supplementary Note 1. TabPFN fine-tuning with LQR indicators.

We performed cross-national fine-tuning of the default TabPFN weights using only the LQR predictors (LQR and survey language) to examine their impact on predictive performance. Fine-tuning led to modest but consistent improvements across independent test datasets, with the greatest improvements observed at the smallest fine-tuning training proportion (25%). Specifically, when the fine-tuned weights were applied to the cross-national models, improvements occurred in 92.6% of questionnaires in the four-country dataset (using LQR, survey language, and demographics), rising to 96.3% when activity features were included. In external validation on CHARLS, improvements were observed across all questionnaires for both models. The largest single improvement was a 4.65% (AUROC increase = 0.033) gain in the MHAS Locus of Control – Luck questionnaire (Supplementary Table 5).

Further, the pattern of improvement was generally aligned with our earlier observation in the complexity–response quality analysis: that is, there were greater fine-tuning benefits for questionnaires characterised by higher complexity and lower overall response quality, and this trend was more pronounced in the HRS.

Supplementary References

1. Hardy, S. E., Allore, H. & Studenski, S. A. Missing Data: A Special Challenge in Aging Research: MISSING DATA. *Journal of the American Geriatrics Society* **57**, 722–729 (2009).
2. Knäuper, B., Belli, R. F., Hill, D. H. & Herzog, A. R. Question Difficulty and Respondents' Cognitive Ability: The Effect on Data Quality. *Journal of Official Statistics* **13**, (1997).
3. Graham, J. W. Missing data analysis: making it work in the real world. *Annu Rev Psychol* **60**, 549–576 (2009).
4. Krosnick, J. A. Response strategies for coping with the cognitive demands of attitude measures in surveys. *Applied Cognitive Psychology* **5**, 213–236 (1991).
5. Muthén, L. K. & Muthén, B. O. *Mplus: Statistical Analysis with Latent Variables: User's Guide*. (Muthén & Muthén, Los Angeles, CA, 2017).
6. Meijer, R. R. Diagnosing item score patterns on a test using item response theory-based person-fit statistics. *Psychol Methods* **8**, 72–87 (2003).
7. Meijer, R. R. The Number of Guttman Errors as a Simple and Powerful Person-Fit Statistic. *Applied Psychological Measurement* **18**, 311–314 (1994).
8. Tendeiro, J. N., Meijer, R. R. & Niessen, A. S. M. PerFit: An R Package for Person-Fit Analysis in IRT. *J. Stat. Soft.* **74**, 1–27 (2016).
9. Emons, W. H. M. Nonparametric Person-Fit Analysis of Polytomous Item Scores. *Applied Psychological Measurement* **32**, 224–247 (2008).
10. Tabachnick, B. G. & Fidell, L. S. *Using Multivariate Statistics (4th Ed.)*. (Allyn and Bacon, 2001).
11. Conijn, J. M., van der Ark, L. A. & Spinhoven, P. Satisficing in Mental Health Care Patients: The Effect of Cognitive Symptoms on Self-Report Data Quality. *Assessment* **27**, 178–193 (2020).
12. Meade, A. W. & Craig, S. B. Identifying careless responses in survey data. *Psychological Methods* **17**, 437–455 (2012).
13. Schneider, S. Extracting Response Style Bias From Measures of Positive and Negative Affect in Aging Research. *J Gerontol B Psychol Sci Soc Sci* **73**, 64–74 (2017).
14. Falk, C. F. & Cai, L. A flexible full-information approach to the modeling of response styles. *Psychological Methods* **21**, 328–347 (2016).
15. Falk, C. F. & Ju, U. Estimation of response styles using the multidimensional nominal response model: A tutorial and comparison with sum scores. *Frontiers in Psychology* **11**, (2020).
