# Supplementary material for: Low-burden AI approach for cross-national early identification of cognitive impairment using real-world questionnaire response behaviours
Source: Nat Commun. 2026 Jul 28;17:9157. doi: 10.1038/s41467-026-76071-9 (PMC13522467; doi:10.1038/s41467-026-76071-9)
Supplement: Supplementary file 2 [file 41467_2026_76071_MOESM2_ESM.pdf]

nature portfolio

Corresponding author(s): Hongxin Gao

Last updated by author(s): Jun 11, 2026

Reporting Summary

Nature Portfolio wishes to improve the reproducibility of the work that we publish. This form provides structure for consistency and transparency in reporting. For further information on Nature Portfolio policies, see our Editorial Policies and the Editorial Policy Checklist.

Statistics

For all statistical analyses, confirm that the following items are present in the figure legend, table legend, main text, or Methods section.

n/a	Confirmed	
☐	☒	The exact sample size (*n*) for each experimental group/condition, given as a discrete number and unit of measurement
☐	☒	A statement on whether measurements were taken from distinct samples or whether the same sample was measured repeatedly
☐	☒	The statistical test(s) used AND whether they are one- or two-sided<br>*Only common tests should be described solely by name; describe more complex techniques in the Methods section.*
☐	☒	A description of all covariates tested
☐	☒	A description of any assumptions or corrections, such as tests of normality and adjustment for multiple comparisons
☐	☒	A full description of the statistical parameters including central tendency (e.g. means) or other basic estimates (e.g. regression coefficient) AND variation (e.g. standard deviation) or associated estimates of uncertainty (e.g. confidence intervals)
☐	☒	For null hypothesis testing, the test statistic (e.g. *F*, *t*, *r*) with confidence intervals, effect sizes, degrees of freedom and *P* value noted<br>*Give P values as exact values whenever suitable.*
☒	☐	For Bayesian analysis, information on the choice of priors and Markov chain Monte Carlo settings
☐	☒	For hierarchical and complex designs, identification of the appropriate level for tests and full reporting of outcomes
☒	☐	Estimates of effect sizes (e.g. Cohen's *d*, Pearson's *r*), indicating how they were calculated

Our web collection on statistics for biologists contains articles on many of the points above.

Software and code

Policy information about availability of computer code

Data collection	No specialized software or custom code was used for data collection.
Data analysis	Data analysis was performed on a cloud-based interactive computing platform (Intel Xeon CPU, 2.20 GHz, 12 cores; NVIDIA A100 GPU, 40GB, CUDA 12.4), using Python (versions 3.12.11 for general analyses and 3.11.13 for fine-tuning experiments) and R (version 4.2.1). Key Python packages include TabPFN (v2.0.9), XGBoost (v2.1.4), LightGBM (v4.5.0), CatBoost (v1.2.8), SHAP (v0.48.0), NumPy (v2.0.2), pandas (v2.2.2), PyTorch (v2.6.0), scikit-learn (v1.5.2), scipy (v1.15.3), matplotlib (v3.10.0), and seaborn (v0.12.2). Custom code, detailed software packages, and model weights are publicly available via GitHub at: https://github.com/ocean8800v/cross-national-cognitive-ai and archived on Zenodo at https://doi.org/10.5281/zenodo.20651481.

For manuscripts utilizing custom algorithms or software that are central to the research but not yet described in published literature, software must be made available to editors and reviewers. We strongly encourage code deposition in a community repository (e.g. GitHub). See the Nature Portfolio guidelines for submitting code & software for further information.

Data

Policy information about availability of data

All manuscripts must include a data availability statement. This statement should provide the following information, where applicable:

- Accession codes, unique identifiers, or web links for publicly available datasets
- A description of any restrictions on data availability
- For clinical datasets or third party data, please ensure that the statement adheres to our policy

The datasets analysed in this study are from five ageing cohorts accessed through registration or application procedures: the Health and Retirement Study (HRS), the English Longitudinal Study of Ageing (ELSA), the Longitudinal Aging Study in India (LASI), the Mexican Health and Aging Study (MHAS), and the China Health and Retirement Longitudinal Study (CHARLS), including relevant Harmonized Cognitive Assessment Protocol (HCAP) data. These cohort data are not owned by the authors and cannot be redistributed by the authors. They are available under controlled access through the Gateway to Global Aging Data (https://g2aging.org/) and the relevant cohort data portals, subject to registration, application where required, and the applicable data-use agreements. Access requests are handled by the relevant data providers according to their own access procedures, eligibility criteria, response timelines and data-use terms. Participant-level derived low-quality response indicators are based on these controlled-access cohort questionnaire data and are therefore subject to the original cohort data-use agreements rather than deposited separately. Source data are provided with this paper.

Research involving human participants, their data, or biological material

Policy information about studies with human participants or human data. See also policy information about sex, gender (identity/presentation), and sexual orientation and race, ethnicity and racism.

Reporting on sex and gender	Sex (male/female) data were available for all participants and used in analyses (overall: 47.3% male, 52.7% female). Sex was determined based on self-report. Sex-based subgroup analyses of model discrimination were performed and are reported in the manuscript. Gender (shaped by social and cultural circumstances) was not explicitly analysed in this study, as the original cohort datasets — the Health and Retirement Study (HRS), English Longitudinal Study of Ageing (ELSA), Longitudinal Ageing Study in India (LASI), Mexican Health and Ageing Study (MHAS), and China Health and Retirement Longitudinal Study (CHARLS) — did not explicitly distinguish gender from biological sex.
Reporting on race, ethnicity, or other socially relevant groupings	Race and ethnicity were not analysed in this study.
Population characteristics	Secondary data from nationally representative ageing cohorts (HRS, ELSA, LASI, MHAS, CHARLS); all participants aged ≥65 years.
Recruitment	Secondary analysis of controlled-access ageing cohort datasets; no primary recruitment was conducted for this study.
Ethics oversight	This study complies with all relevant ethical regulations. Ethical approval for the original cohort data collection was obtained from the following institutions: HRS (approved by the University of Michigan Institutional Review Board); ELSA (approved by the South Central-Berkshire Research Ethics Committee, 13/SC/0532 and 15/SC/0526); LASI (approved by the Indian Council of Medical Research, Delhi, and the Institutional Review Board of the International Institute for Population Sciences, Mumbai); MHAS (approved by the institutional review boards of the University of Texas Medical Branch and the Instituto Nacional de Estadística y Geografía, Mexico); and CHARLS (approved by the Biomedical Ethics Review Committee of Peking University, IRB00001052-11015). All participants in the original cohort studies provided written informed consent. This study involves secondary analysis of de-identified, controlled-access data and was approved by the University of Surrey Research Integrity and Governance Office (FHMS 21-22 216 EGA) and the University of Southern California Institutional Review Board (UP-22-00147).

Note that full information on the approval of the study protocol must also be provided in the manuscript.

Field-specific reporting

Please select the one below that is the best fit for your research. If you are not sure, read the appropriate sections before making your selection.

☐ Life sciences ☒ Behavioural & social sciences ☐ Ecological, evolutionary & environmental sciences

For a reference copy of the document with all sections, see nature.com/documents/nr-reporting-summary-flat.pdf

Behavioural & social sciences study design

All studies must disclose on these points even when the disclosure is negative.

Study description	Quantitative study developing an AI pipeline for early identification of cognitive impairment risk based on behavioural response metrics from population-based ageing cohorts.

Research sample	45,604 older adults aged ≥65 years (52.7% female) from five population-based ageing cohorts: HRS (US), ELSA (England), LASI (India), MHAS (Mexico), and CHARLS (China). The cohorts are nationally representative ageing studies in their respective countries. Data were accessed through controlled-access data platforms, including the Gateway to Global Aging Data (https://g2aging.org/) and relevant cohort data portals. Sex/gender was cohort-reported and included in the study design as a demographic variable. Participants provided written informed consent during the original cohort data collection; participant compensation was determined by each original cohort study.
Sampling strategy	Participants from five controlled-access ageing cohorts (HRS, ELSA, LASI, MHAS, CHARLS) were included if they: (1) completed psychosocial questionnaires during each country's Harmonised Cognitive Assessment Protocol (HCAP) wave; (2) were aged ≥65 years at the HCAP wave; and (3) participated via non-proxy interviews. No a priori sample-size calculation was performed; all 45,604 eligible respondents were included.
Data collection	Secondary controlled-access datasets were accessed through the Gateway to Global Aging Data platform (https://g2aging.org/) and relevant cohort data portals, subject to registration, application where required, and applicable data-use agreements.
Timing	Primary analyses used cross-sectional data from cohort waves corresponding to each country's HCAP wave: HRS Wave 13 (2016), ELSA Waves 7–8 (2014–2017), LASI Wave 1 (2017–2019), MHAS Wave 4 (2015), and CHARLS Wave 4 (2018). Prospective validation used earlier-wave data where available: HRS Wave 11, ELSA Wave 6, MHAS Wave 3, and CHARLS Wave 3.
Data exclusions	Participants aged <65 years, those who did not complete the relevant psychosocial questionnaires, or those with proxy interviews were excluded based on pre-established eligibility criteria.
Non-participation	This study is a secondary analysis of existing controlled-access cohort data; response rates and reasons for non-participation at the original recruitment stage were determined by each contributing cohort study (HRS, ELSA, LASI, MHAS, CHARLS) and are reported in their respective methodological documentation. No primary recruitment was conducted for the present analysis.
Randomization	This was an observational study using existing survey data; therefore, randomisation was not part of the study design.

Reporting for specific materials, systems and methods

We require information from authors about some types of materials, experimental systems and methods used in many studies. Here, indicate whether each material, system or method listed is relevant to your study. If you are not sure if a list item applies to your research, read the appropriate section before selecting a response.

Materials & experimental systems

n/a	Involved in the study	
☒	☐	Antibodies
☒	☐	Eukaryotic cell lines
☒	☐	Palaeontology and archaeology
☒	☐	Animals and other organisms
☒	☐	Clinical data
☒	☐	Dual use research of concern
☒	☐	Plants

Methods

n/a	Involved in the study	
☒	☐	ChIP-seq
☒	☐	Flow cytometry
☒	☐	MRI-based neuroimaging

Plants

Seed stocks	*Report on the source of all seed stocks or other plant material used. If applicable, state the seed stock centre and catalogue number. If plant specimens were collected from the field, describe the collection location, date and sampling procedures.*
Novel plant genotypes	*Describe the methods by which all novel plant genotypes were produced. This includes those generated by transgenic approaches, gene editing, chemical/radiation-based mutagenesis and hybridization. For transgenic lines, describe the transformation method, the number of independent lines analyzed and the generation upon which experiments were performed. For gene-edited lines, describe the editor used, the endogenous sequence targeted for editing, the targeting guide RNA sequence (if applicable) and how the editor was applied.*
Authentication	*Describe any authentication procedures for each seed stock used or novel genotype generated. Describe any experiments used to assess the effect of a mutation and, where applicable, how potential secondary effects (e.g. second site T-DNA insertions, mosiacism, off-target gene editing) were examined.*
